# Rodent Models of Audiogenic Epilepsy: Genetic Aspects, Advantages, Current Problems and Perspectives

**DOI:** 10.3390/biomedicines10112934

**Published:** 2022-11-15

**Authors:** David G. Garbuz, Artem A. Davletshin, Svetlana A. Litvinova, Irina B. Fedotova, Natalya M. Surina, Inga I. Poletaeva

**Affiliations:** 1 Laboratory of Molecular Mechanisms of Biological Adaptation, Engelhardt Institute of Molecular Biology of the Russian Academy of Sciences, 119991 Moscow, Russia; 2 Laboratory of Psychopharmacology of FSBI “Zakusov Institute of Pharmacology”, 125315 Moscow, Russia; 3 Chair of Higher Nervous Activity, Biology Department, Lomonosov Moscow State University, 119991 Moscow, Russia

**Keywords:** audiogenic epilepsy, genetic epilepsy models, epilepsy-associated genes, KM rats, WAR rats, GASH/Sal hamster strain, epileptogenesis

## Abstract

Animal models of epilepsy are of great importance in epileptology. They are used to study the mechanisms of epileptogenesis, and search for new genes and regulatory pathways involved in the development of epilepsy as well as screening new antiepileptic drugs. Today, many methods of modeling epilepsy in animals are used, including electroconvulsive, pharmacological in intact animals, and genetic, with the predisposition for spontaneous or refractory epileptic seizures. Due to the simplicity of manipulation and universality, genetic models of audiogenic epilepsy in rodents stand out among this diversity. We tried to combine data on the genetics of audiogenic epilepsy in rodents, the relevance of various models of audiogenic epilepsy to certain epileptic syndromes in humans, and the advantages of using of rodent strains predisposed to audiogenic epilepsy in current epileptology.

## 1. Introduction: The Relevance of the Problem

Epilepsy is a heterogeneous group of chronic neurological diseases characterized by recurrent seizure fits. It is one of the most common neurological problems of our time. According to WHO, the incidence of epilepsy varies from 4 to 14 cases per 1000 people in different countries. Being a life-threatening disease, epilepsy leads to a significant reduction in quality of life as well. Severe cases may be accompanied by numerous complications: seizure induced injuries, cerebral ischemia, cardiogenic shock, headaches, intellectual disability, psychosis, hallucinations, depression, and status epilepticus (prolonged seizures or series of seizures without recovery of consciousness between them) [1,2,3,4,5,6].

Epilepsy can develop as an independent disease or could be a consequence of other pathologies, such as brain developmental disorders, ischemic stroke, neurodegenerative diseases, traumas, infections, etc. [7,8]. Today, the leading role in the pathogenesis of most forms of epilepsy is recognized to have a genetic component. More than 1000 genes are known whose mutations are associated with the development of epilepsy [9,10]. These data, as well as the growing understanding of the molecular mechanisms of various mutations that affect neuronal activity in different brain regions, open up prospects for a targeted impact on the pathogenesis of this disease. A large number of antiepileptic drugs (AEDs) and clinical protocols for epilepsy drug treatment have been developed, but there is also high pharmacoresistance (up to 30% of epilepsy cases) [11]. In addition, many AEDs are characterized by severe side effects as well [12,13,14,15], and this requires the search for new, more effective and less toxic anticonvulsants.

The study of epilepsy mechanisms and the development of new methods of its treatment, including the discovery of new pharmacological targets, require adequate animal models. Numerous experimental techniques of epileptic seizures and status epilepticus induction were introduced, including the electrical stimulation of certain brain regions or infusion or application of convulsants, i.e., substances that provoke the development of seizures [16,17,18,19]. However, most of these approaches do not allow studying the genetic causes of epilepsy. For this reason, a large number of investigations are focused on deriving animal strains with a genetic predisposition to seizures, which could be either the result of directed genetic knockouts or classical selection experiments [20,21,22,23,24,25]. Models of inherited audiogenic epilepsy (AE) described in several rodent species stand out favorably among such strains. Their advantages are the stable seizure pattern, easy seizure induction without invasive influences, easily visualized seizure, thus requiring no prolonged observation and/or EEG recording to identify seizure occurrence (procedure inherent for spontaneous seizure models), and high reproducibility of results inherent as well. Based on AE models, it is possible both to search for new genes involved in the development of seizure states and to test new AEDs [26].

In this paper, we attempt to systematize data on the genetic mechanisms associated with AE in rodents and evaluate the possibility of applying the results to human epilepsy science. The applicability of AE models for testing new anticonvulsant compounds as well as for the basic research of epilepsy molecular mechanisms will be assessed.

## 2. Classification and Etiology of Epilepsy

Epilepsy is a heterogeneous group of syndromes that differ significantly from each other both in the clinical manifestations and in the etiology of the disease. The International Classification of Epilepsy and Epileptic Syndromes was adopted in 1989 and was subsequently revised by The International League Against Epilepsy (ILAE) [27]. Currently, human epilepsy is classified according to the “Revised terminology and concepts for organization of seizures and epilepsies: report of the ILAE Commission on Classification and Terminology” [27].

According to the updated version of epilepsy classification, each particular case is considered at several diagnostic levels. The first one is the type of seizure, the second is the type of epilepsy (focal, generalized, combined focal-generalized, and unclassified), followed by the definition of the epileptic syndrome, and finally, the etiology of epilepsy. The main manifestation of epilepsy is seizures that are divided into atonic (loss of consciousness and muscle tone and subsequent rapid recovery), clonic (irregular short-term spasms characterized by a rapid change in periods of contraction and relaxation of skeletal muscles), tonic (prolonged tension of the muscles of the limbs or the entire body, including respiratory muscles), and myoclonic (rapid contraction of various muscle groups). There are also non-convulsive epileptic seizures (absences) in the form of a momentary loss of consciousness [28].

Depending on the degree of brain structure involvement, there are focal, generalized, and combined forms of epilepsy. In focal epilepsy (e.g., temporal lobe epilepsy), a local epileptic focus is formed in one of the hemispheres of the brain, most often as a result of the formation of focal pathology (stroke, tumor) or trauma. In generalized epilepsy, an abnormal excitation spreads widely in the brain; in this case, seizures are associated with the simultaneous activation of many areas of both hemispheres [29,30]. In the case of a combined form of epilepsy (e.g., Dravet syndrome), both focal and generalized seizures may be recorded [31].

The next level of classification identifies the type of epileptic syndrome as a set of characteristics (seizure type, EEG and neuroimaging data, and comorbidity traits) [32]. In some cases, a seizure is provoked by clearly identifiable specific triggers that could be external (visual, vestibular, auditory, or tactile stimuli) or internal stimuli (elementary or higher neural functions such as certain movements, emotions, or calculations). In this case, one can identify a reflex form of epilepsy. Reflex epileptic seizures could be focal or generalized, and in some cases occur along with spontaneous seizures [33].

The next stage of classification establishes the etiology of the disease. According to the new classification, all cases of epilepsy are divided into structural, genetic, infection-related, metabolic, and immune categories, as well as cases with unknown etiology. Structural epilepsies are determined by a marked defect in brain morphology (due to developmental abnormality, trauma, or stroke) [7,8]. Thus, structural epilepsies can be either genetic (with severe CNS developmental abnormalities due to mutations) or acquired. Genetic epilepsies are caused by mutations that are not accompanied by significant anomalies in CNS development and morphology but cause disruptions in brain cell functions (e.g., mutations of ion channel genes) [34,35]. Metabolic epilepsies are the direct result of a known or suspected metabolic disorder, with epilepsy dominating the clinical picture. They are often well-known inherited metabolic diseases, such as porphyria, amino acid metabolism disorders, creatine disorders, and pyridoxine-dependent seizures. Thus, metabolic epilepsy in most cases can also be determined by genetic factors [36,37]. Infectious epilepsies develop as a consequence of past or chronic neuroinfections, including intrauterine (neurocysticercosis, tuberculosis, HIV, viral encephalitis, toxoplasmosis, Zika fever, and COVID-19) [38,39,40,41]. Finally, immune (or autoimmune) epilepsies develop as a direct consequence of autoimmune processes. An example is encephalitis with the detection of autoantibodies to NMDA receptors [42,43,44]. In some cases, we can run into cases of the combined form of epilepsy. For example, some infectious agents often induce autoimmune processes that lead to structural brain damage with epilepsy as clinical manifestations (in such cases, epilepsy combines infectious, autoimmune, and structural etiologies) [45,46,47]. Thus, the new classification of epilepsy allows highlighting the main cause of the disease: purely genetic, genetic in combination with structural disorders, structural disorders due to non-genetic causes, etc.

## 3. Pathophysiological Mechanisms of Epilepsy

At present, the molecular mechanisms of increased neuronal excitability in epilepsy are known in general terms and require further study for picture refinement. The immediate cause of epileptogenesis is the increased excitability of neuronal groups due to the imbalance of excitatory and inhibitory neurotransmitter systems. Such disturbances lead to a prolonged depolarization of the neuronal membrane, which causes a decrease in the threshold for action potentials and massive activation, spreading from the primary epileptic focus to other CNS structures.

The main changes in excitatory systems during epileptogenesis affect the glutamatergic system. The excessive activation of ionotropic NMDA and AMPA glutamate receptors is the one of most common causes of epileptogenesis, which occurs during and after an epileptic seizure. A quantitative increase in ionotropic NMDA receptors in the brain of epileptic patients and in many epilepsy animal models has been demonstrated. This change could also be accompanied by a shift in the subunit composition of receptor proteins, mainly due to an increase in calcium-permeable subunits [48,49]. The characteristics of type II metabotropic glutamate receptors (mGluRII) located on the presynaptic membrane and controlling the activity of glutamatergic synapses could also be affected [50,51,52]. Quantitative and qualitative rearrangements of ionotropic and metabotropic glutamate receptors lead to higher intensity of glutamatergic signaling, increasing seizure readiness.

The dopaminergic system is involved in the regulation of glutamatergic synaptic transmission activity through modulation of mitogen-activated kinase (ERK1/2) activity (part of MAPK signaling pathway) [53,54,55]. ERK1/2 stimulates the expression of NMDA receptors and some other synaptic proteins, which increases synaptic excitability [56,57]. In addition, ERK1/2 phosphorylates synapsin I, promoting the release of glutamate from presynaptic vesicles [58]. The stimulation of D1 receptors causes the phosphorylation and activation of ERK1/2, whereas the activation of the D2/D3-dependent pathway has the opposite effect [59,60,61]. Thus, a shift in the balance of dopamine receptors toward an increase in D1 receptors leads to increased neuronal excitability, which has been observed in epileptic patients and model animals [62,63,64]. In addition to the glutamatergic and dopaminergic systems, the other neurotransmitter systems were also shown to be affected in epileptogenesis development [65,66,67,68,69,70,71].

The decrease in neuronal excitability that has antiepileptic effects is based mainly on the GABAergic system function. In cases of epilepsy development, the decrease in the number of GABA_A_ receptors as well as changes in their subunit composition, including those involving ERK1/2, has been demonstrated both in humans and in animal models [72]. In some cases, decreased GABA production and/or increased GABA reuptake in synapses have been shown [73,74,75,76,77,78].

In addition to the imbalance of neurotransmitter systems, changes in the activity and/or amount of potential-dependent ion channels, in particular, Na-, K- and Ca channels, play an important role in epileptogenesis, leading to the increased entry of Na^+^ and Ca^2+^ ions into the cell and K^+^ ions exiting from the cell [35,79,80,81]. These changes decrease the voltage of membrane potential and the threshold of neuronal excitation, leading to the generation of the action potential. Increased intracellular Ca^2+^ concentration could also lead to the activation of glutamate release, decreased GABA levels, and activation of proinflammatory signaling cascades [81,82]. Together with neuronal excitation changes, an important role in epileptogenesis is now ascribed to glia. Reactivated astrocytes, in particular, increase the concentration of glutamate and K^+^ ions in the nervous system, contributing to the spread of neuronal excitation [83]. Unfortunately, even a brief description of this issue is beyond the scope of this review. To get acquainted with the problem, we can recommend other reviews [84,85].

Thus, we can summarize that one of the main causes of epilepsy is disturbance in the normal activity of ion channels (both receptor- and potential-dependent). These disorders may result from mutations in the genes encoding the respective ion channels, changes in the signal cascades activity, affecting their expression, metabolic disorders (as a result of mutations in enzyme genes), mutations leading to the abnormal development of the nervous system resulting in the abnormally high activity of “excitatory” neurons and decreased activity of “inhibitory” ones, and/or in excessive astrogliosis [86]. Neuroinflammation leading to astrocyte activation may also play an important role in the development of epilepsy [87,88]. The changes described initiate the development of an epileptic focus, i.e., a pool of neurons with prolonged membrane depolarization. Due to the diffusion of glutamate and potassium ions into the intercellular space, the excitation thresholds of the adjacent neurons also decrease, and the excitation of such neuronal pool synchronizes. The excitation, born in the primary epileptogenic focus, spreads to other parts of the brain, and the dynamic epileptic system with the development of secondary epileptogenic foci emerges, which in turn can acquire determinant properties [89].

Below, several genetic causes are analyzed concerning the changes in neuronal excitability that may lead to the development of epilepsy.

## 4. Genetic Aspects of Human Epilepsy: Brief Review

A significant number of epilepsy cases could be considered inherited diseases with the possibility of identifying mutations responsible for the development of seizures [9,10,90]. Genetic variants sometimes could be identified, which influence the likelihood of epileptic seizures emergence in cases of focal brain lesions (strokes, tumors) [10].

Broad-scale epidemiological studies provide convincing evidence for the important role of the genetic factor: the risk of developing epilepsy in patients with a family history of seizure disorders in close relatives is 8 to 12%, which is significantly higher than the general population’s respective average risk (approximately 0.5 to 1%) [91,92,93,94]. To date, about 1000 genes associated with the development of various forms of epilepsy have been identified [9,10]. Some forms of hereditary epilepsy are monogenic, being inherited both by autosomal dominant (e.g., sodium channel alpha-subunit *SCN1A* gene mutations in Dravet syndrome or *familial febrile seizures*) [95] and autosomal recessive types (in case of *TBC1D24* gene mutation in *familial infantile myoclonic epilepsy*) [96]. There are forms of epilepsy and complex diseases that include seizures as one of the symptoms that are inherited as X-linked in a dominant or recessive type (*CDKL5* gene mutations in *early infantile epileptic encephalopathy* or *SYN1* in *X-linked epilepsy with variable learning disabilities and behavior disorders* and some forms of refractory epilepsy) [97,98,99]. However, most cases of epilepsy appear to have a polygenic basis and are determined by a combination of many specific alleles of certain genes associated with CNS development in the prenatal and early postnatal periods [9,10,100,101,102,103]. Even in cases of the monogenic inheritance of epilepsy, the severity and the pattern of epileptic seizures may depend largely on the genetic background [9]. Individual cases, diagnosed as the same form of epilepsy, very often happen to be caused by different single mutations or combinations of many “unfortunate” alleles. For example, in *early infantile epileptic encephalopathy* (EIEE), more than 40 genes may be associated with the development of the disease [9]. On the other hand, mutations in the same gene could be found in different epilepsy phenotypes; for example, mutations in the *GABRA1* gene are associated with EIEE, *childhood absence epilepsy* (CAE) and *juvenile myoclonic epilepsy* (JME) [104,105,106]. Finally, many genetic variants do not lead to epilepsy in 100% of cases but are expressed in cases of combination with adverse external conditions and/or traumatic influences (stress, sleep deprivation, stroke, and infections) [107]. In some cases, mutations leading to the development of epilepsy occur *de novo*, i.e., they were absent in parents but found in a child, sometimes in the form of chimerism (due to a somatic mutation that occurred early in embryogenesis) [108]. Thus, it is extremely difficult to systematize the genetic causes of epilepsy, both because of the diversity of its clinical manifestations and because of the many combinations of genetic variants that can lead to different results in terms of certain forms of epilepsy development.

In general, the diversity of genes associated with epilepsy can be divided into several large functional groups [9,10,109]. A significant percentage of epilepsy cases with autosomal dominant inheritance are due to mutations in genes encoding various subunits of potential-dependent and receptor ion channels: sodium, potassium, calcium, chlorine, GABA_A_-receptor subunits, NMDA-receptors and acetylcholine receptors. Sometimes, such cases are grouped under the general name “channelopathies”. Mutations of these genes directly lead to excitation/inhibition imbalance in the central nervous system and, as a consequence, to the development of epileptic seizures. The next groups of genes associated with the development of epilepsy are those encoding enzymes and enzyme activity-regulating proteins [110,111,112,113]. Finally, the less-represented groups include genes responsible for cell adhesion, signal transduction proteins, membrane trafficking proteins, and cytoskeletal proteins. In addition, in some cases, mtDNA mutations that disrupt the functions of mitochondrial proteins and tRNAs can lead to the development of epilepsy [114,115].

**Figure 1:**
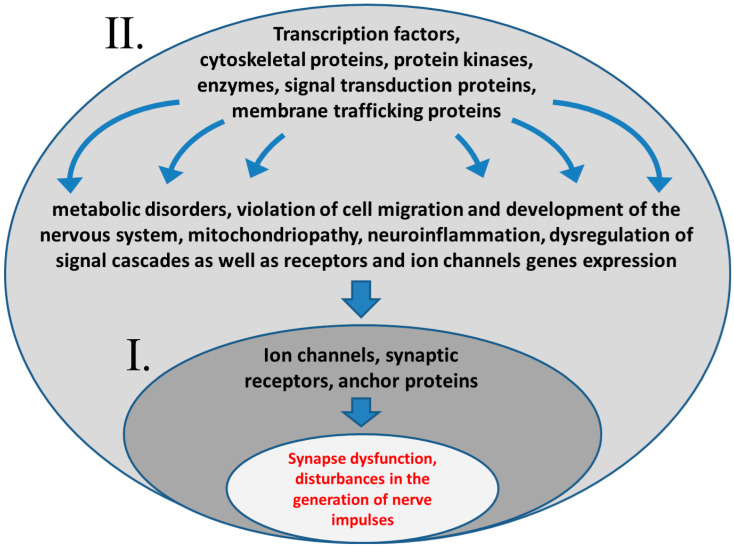
Mutations leading to the development of epilepsy may affect genes directly involved in the generation and transmission of nerve impulses (group I), or genes responsible for the development and metabolism of the nervous system (group II). In the second case, violations of the functions of ion channels and specific synaptic proteins occur indirectly.

Disruption in these gene functions can lead to the development of epilepsy in different ways (Figure 1). Mutations in genes encoding subunits of potential-dependent and receptor ion channels directly lead to the persistent depolarization of neuronal membranes and, consequently, to their epileptization. Such disorders (channelopathies) are responsible for approximately 15% of epilepsy cases [83,116]. Disorders in the functions of receptor genes, signal transduction proteins and membrane trafficking proteins, cytoskeleton proteins, and mitochondrial enzymes initiate a more complex chain of events. Such disorders can lead to a shift in the balance of neurotransmitters, or indirectly lead to the development of structural or metabolic epilepsy, which could be the consequences of developmental anomalies of the brain, metabolic syndromes, or neurodegeneration [113]. A direct cause of these types of epilepsy could lie, particularly, in changes of signaling cascades activity, including those regulating neurotransmitter release and ion channel gene expression (e.g., MAPK and mTOR) [10,57,117,118,119,120]. The imbalance in signaling cascades activity can lead, also, to cell cycle deteriorations during neuronal proliferation; the development of mitochondriopathy; excessive apoptosis, affecting definite types of neurons; the development of neuroinflammation; and other mechanisms of epileptogenesis, which has been mainly studied in animal models [121,122,123,124,125,126]. Thus, some cases of genetically determined epilepsy are the result of disturbances in the complex networks regulating the development and homeostasis of the nervous system.

## 5. Animal Models of Epilepsy

Creating experimental models of epilepsy in animals (mainly in rodents) is the main way to study the pathophysiological mechanisms of seizure development and to search for new targets of new AEDs. There are many such models available today, but no single model can fully capture all the features of epilepsy and describe all the variety of symptoms observed in humans [127].

Traditionally, the most widely used models are those in which epileptic seizures in mice or rats are induced either by repeated stimulation of various brain structures with an electric current or by administration of pharmacological drugs that decrease the seizure barrier. Such exposure has been called “kindling”. Kindling is a phenomenon in which, in response to repeated epileptogenic stimuli of subthreshold intensity, the convulsive threshold of the brain decreases, and spontaneous convulsions consistently develop. The kindling process suggests that it starts with a limited number of neural circuits and subsequently recruits additional circuits as the behavioral component of the seizure progresses to seizures [128]. The classical variant of electrical kindling is the repeated subliminal electrical stimulation of limbic structures of the brain, which results in the development of epileptic seizures. Pharmacological kindling involves repeated exposure to subthreshold doses of convulsants, such as pentylentetrazole (PTZ) or kainate, resulting in the development of spontaneous seizures. Kindling models allow the induction of spontaneously recurrent seizure development, as commonly seen in patients with temporal lobe epilepsy [129]. The historically more “old” chronic epilepsy model is cobalt-induced epilepsy [130,131]. The rocking of the epileptic system also occurs after a powerful single epileptogenic exposure, such as pilocarpine or kainate, inducing status epilepticus (post-status models). Post-status patterns are most consistent with refractory forms of epilepsy because they are accompanied by neuronal death, aberrant neurogenesis, development of encephalopathy, etc. [132].

The “gold standard” in the search for new potentially active (AEDs) are simple models of acute seizures: the maximal electroshock test (MES) and subcutaneous injection of PTZ in mice and rats. These models have been used for the testing and discovery of most potential AEDs [133,134]. The advantage of these epilepsy models is that they do not require special strains of laboratory animals. Their disadvantage is that they do not reflect such an important aspect as the contribution of genetic factors in epilepsy development.

Therefore, genetic models of seizures, i.e., animal strains with an innate predisposition to epilepsy, are widely used in laboratory practice. These models make it possible to study the contribution of individual genes, groups of genes, and related signaling cascades in epilepsy development, which is necessary to find new AEDs targets. Today, there are genetic strains derived from several animal species (baboons, chickens, rats, mice, hamsters, and felines) that are seizure-prone (both spontaneous and refractory convulsions). For example, the seizure response in *Papio papio* baboons and Fepi chicken strains could be induced by rhythmic flashes of light, and in cats and many rodent strains by a sudden loud sound (audiogenic epilepsy, AE) [26]. Photosensitive epilepsy is quite common in epileptic patients, whereas audiogenic seizures in humans are very rare; their closest analogs in humans are startle-induced seizures [26]. Nevertheless, it is the AE-prone rodents that are commonly used as epilepsy models in laboratory practice because their reaction to a seizure-provoking stimulus (sound) is easily reproduced, and they display the standard type of seizure fit. Standard and experimental AEDs reduce the intensity of seizures in AE-prone rodents, and thus these strains could be used for the search for new AEDs and analysis of their anticonvulsive mechanisms [26,135]. Therefore, rodent AE-prone strains can generally be considered to be sufficiently fit for these studies, not only for human reflex epilepsy.

Genetic abnormalities in rodent AE-prone strains and in human epilepsy may coincide, but in some cases, they demonstrate differences as well (see below). On the other hand, as mentioned earlier, even in epilepsy clinics, a huge variety of both symptomatic manifestations and their genetic causes could be found in different patients.

Historically, work on genetic models of epilepsy in rodents began in the early 1900s, when the outbred strain of albino Wistar rats (existing till now) was derived in the Wistar Institute (Philadelphia, PA, USA). A certain percentage of these animals developed epileptiform seizures in response to loud sounds [21]. Different sources indicate different percentages of Wistar rats sensitive to sound, from 15 to 50%; apparently, sensitivity varies considerably in different Wistar substrains maintained in isolation in different catteries for many years [121,136]. Subsequently, a similar phenomenon of AE-proneness was found in mice [137,138]. In general, one may conclude that many laboratory rodent strains are generally characterized by increased sensitivity to loud sounds, which rodents do not encounter in a natural habitat, and which is presumably the hypertrophied startle reaction in response to alarming stimuli [139]. Therefore, the selection of strains predisposed to AE in rodents is rather quick, as rodent CNS has presumably the “stereotyped pattern” of reaction to strong sound. Thus, mutation events, affecting different genes in such a cascade, enhance the reaction up to the pathological level. A wide set of rodent strains (including rats, mice, and hamsters) with genetically determined AE were obtained. Animals of these strains respond to loud sounds by displaying generalized seizures. In all AE-prone strains, seizures proceed according to a similar pattern. In response to 100–120 dB sound onset, the first phase of a seizure develops (it is the so-called “wild run stage”), during which the animals rush around the cage or sound chamber. In essence, it is the defense reaction as an attempt to avoid sound, although with an admixture of involuntary movements, indicating the start of convulsions proper (sometimes this stage is called the “clonic run phase”) [139]. The next stages of the seizure proceed: clonic and tonic convulsions, followed by a post-convulsive state (catalepsy, or prolonged excitation) [137,140,141].

The first strain of rats 100% predisposed to audiogenic epilepsy was obtained at Moscow State University by L.V. Krushinsky, L.N. Molodkina and D.A. Fless, which was derived in the late 1940s from the Wistar strain (Krushinsky-Molodkina, abbreviated KM strain) [142]. Independently the WAR (Wistar audiogenic rat) strain was developed at the University of São Paulo, Brazil in the 1990s [25]. In the late 1950s, two GEPR (genetically epilepsy-prone rat) strains, GEPR-3 and GEPR-9, were bred at the University of Arizona based on the Sprague Dawley outbred rat strain, maintained further as independent strains with different levels of AE intensity [143]. The strain of AE-prone rats (P77PMC) also was selected in China [144,145]. The AE-prone hamster strain GASH/Sal (genetic audiogenic seizure hamster, Salamanca) was obtained in the University of Salamanca [146]. There are also several AE-prone mouse strains that were obtained as a result of spontaneous mutations: *Frings*, *DBA/2J*, *Black Swiss*, *101/HY* and *BALB/c* [137,138,147,148]. It has now become a rather common practice to derive AE models by the knockout of genes, presumably associated with epilepsy—they are *Lgi1* mice, *Fmr1* strain, etc. [20,26]. Thus, there is the set of strains with a similar AE phenotype, but with different genetic backgrounds and different genetic causes of this pathology.

Analysis of gene expression profiles and localization of respective mutations as well as the comparisons with each other and with the original AE-non-prone strains could allow revealing both common genetic patterns characteristic of AE and individual strain characteristics, leading to the same result: the development of AE. Using AE-prone strains, it is also possible to compare genetic changes with already established genetic causes of human epilepsy in order to find common mutations and molecular mechanisms leading to seizure proneness. Thus, rodent AE-prone strains increase the scientists’ potential for searching for mutations leading to epilepsy development.

## 6. Pathophysiology of Audiogenic Seizures

Studies of the biochemical peculiarities of AE-prone animals performed in many studies demonstrate numerous “deviations” from the AE-non-prone phenotype practically in all brain neurotransmitter systems tested: glutamatergic, GABAergic, monoaminergic, and purinergic [149]. Numerous studies show that, at the level of biochemical changes, the mechanisms of epileptic seizure in rodents with AE (as in other epilepsy models) and in human epilepsy, in general, are reduced to an imbalance between the glutamatergic and GABAergic systems. In AE-prone rodents, impaired mitochondrial functions, neuroinflammatory processes and increased levels of MAPK signaling cascade activity have also been demonstrated [66,150,151,152].

The well-known similarity of audiogenic seizure fit is based on the similar pattern of brain excitation spreading from the cochlear nuclei up to the corpora quadrigemina (in the case of generalized clonic–tonic seizures) and up to the forebrain structures in the case of audiogenic kindling phenomena, developing after repetitive sound exposures. The AE seizure initiates as the abnormal acoustic impulsation arrives in the *inferior colliculi* (IC). The details of the IC structures’ involvement in seizure initiation have been described in detail with the GEPRs AE model [153,154], as well as in WARs and in mice [155,156,157,158]. Bilateral lesions of the IC, lateral lemniscus and the connections between these structures blocked audiogenic seizures expression in rats and mice [156,157,159]. Further, the *superior colliculi* (SC) activated with the spread of abnormal excitation into brain stem nuclei and further into spinal projections [160]. Rybak and Morin (1995) showed [161] (using immunocytochemistry and in situ hybridization) a significant increase in GABA level and a larger number of GABAergic neurons in the central nucleus of the IC in GEPR-9 strain in comparison to Sprague Dawley AE-non-prone rats. In GEPRs IC, the number of small cells (<15 mcm) was also increased. The IC structure was also affected similarly in KM rats (derived independently from GEPRs). In general, the marked phenotypic similarity in brain structure involved during AE fit is characteristic of rat strains selected in Russia, USA, France and Brazil. The phenomenology of AE seizures as well as numerous data on so-called “priming” procedures in several mouse and rat genotypes were extensively presented in [21]. The role of the acoustic system of AE-prone mouse and rat strains was described in detail earlier [149]. Recently, the signs of acoustic system peculiarities were also demonstrated in GASH/Sal as well [162].

Rodents (rats, mice and Guinea pigs) could be made AE-prone with the injections of metaphit, the drug, which is the ligand of phencyclidine receptors [163,164]. This is another confirmation of the general AE-proneness pattern, and the prevalence of brain excitation circuits. There is experimental evidence suggesting that delta sleep inducing peptide (DSIP) reduced audiogenic seizures, induced by metaphit injections [165]. In general, the effects of this peptide on brain functions are numerous [166,167]. However, the most interesting for the problem of audiogenic seizures mechanism is the evidence of the DSIP influence on the brain antioxidant and glucocorticoid systems [168,169]. It is worth mentioning that the neuronal membrane lipid content in rats of KM strain changed significantly (both quantitatively and qualitatively, in striatum and brain-stem tissues) as the result of audiogenic seizure fit [170]. The data may suggest that the anticonvulsant effect of DSIP was realized via the regulation of membrane processes shifted by metaphit action.

## 7. Different Models of Audiogenic Epilepsy in Rodents

### 7.1. Overview of AE Strains Type

Among AE-prone rodents already described, there are strains with a monogenic inheritance of this trait, and several strains with a presumably polygenic inheritance of the predisposition to AE are now maintained [26]. The new rodent AE strains demonstrate the AE monogenic autosomal dominant or recessive inheritance. They were derived either by targeted genetic knockout or N-ethyl-N-nitrosourea mutagenesis followed by screening for mutations of the target gene [171]. This approach allows the unambiguous association of certain mutations with epilepsy in parallel with data on the sequencing of large epilepsy patient cohorts in search for orthologous gene mutations. There are also rodent strains with monogenic epilepsy derived by subsequent selection after the discovery of respective spontaneous mutations. Strains with a polygenic type of epilepsy inheritance were also obtained independently in different laboratories. These are considered to be the more preferable models of human seizure states since a significant part of epilepsy clinical cases is caused by a combination of multiple “unfortunate” alleles of different genes. At the same time, the localization of specific mutations leading to the development of AE in these strains is a much more difficult task. Brief information about the AE models described in this review is given in Table 1.

### 7.2. Monogenic Models

AE in the mouse strain derived from the *Fmr1* gene knockout was found as a model of human Martin–Bell syndrome (fragile X chromosome syndrome). In patients, this pathology develops as the result of CGG trinucleotide expansion in the X chromosome, which leads to hypermethylation of the promoter region of the *FMR1* gene and a decrease in its expression [172]. The protein product of this gene (fragile X mental retardation protein, FMRP) is responsible for the transport of mRNAs along dendrites and the regulation of the local translation of mRNAs in synapses. The loss of function of this protein leads to the overexpression of glutamate receptors and impaired synaptic plasticity, which in turn results in intellectual disability and autism, macroorchidism, sensory hypersensitivity, and with up to 15% of male and 5% of female patients displaying seizures [173]. Mice with *Fmr1* gene knockout show a phenotype with several similarities to humans: enlarged testes, hyperactivity and mild spatial learning impairment in the Morris water maze, as well as AE [20,174]. The manifestation of audiogenic seizures in fragile X mice is age-dependent. The peak of susceptibility to auditory stimuli in homozygous *Fmr1-/-* females was observed at 22 days of age, after which the intensity of seizures decreased [175]. In another study [176], males of the same genotype showed no convulsions before the age of 10 weeks. At the age of 10–12 weeks, 57% of the fragile X KO mice displayed audiogenic seizures. After the injection of the *Fmr1* transgene, the susceptibility to audiogenic seizures in this strain was reduced [174]. In *Fmr1-/-* mice, seizures were shown to be the result of excitatory metabotropic glutamate receptors and inhibitory GABA_B_ receptors signaling imbalance [177].

In the Frings mice strain, AE is caused by one base deletion at nucleotide 7009 of the *Vlgr1* gene (also known as *GPR98*, *MASS1*, *Neurepin1* and *ADGRV1*), resulting from spontaneous mutation when the truncated protein variant expresses [22,23,24]. The deletion of exons 2 to 4 of the *Vlgr1* gene also leads to the development of AE in mice [23]. The *Vlgr1* gene encodes very large G-protein coupled receptor 1, a member of the adhesion-GPCR (G protein-coupled receptors) family, highly expressed in the embryonic central nervous system [178]. Mutations of the *ADGRV1* (*VLGR1*) gene in humans are associated with the development of myoclonic epilepsy and Usher syndrome [24]. The Vlgr1 protein (referred to by the authors as MASS1) inhibits ubiquitinylation of the myelin-associated glycoprotein (MAG) involving the PKA and PKC signaling cascades [179]. Presumably, the Vlgr1 protein dysfunction (inducing epileptogenesis) is associated with impaired axon myelination. At 3 weeks of age, about 90% of *Vlgr1-/-* mice responded to sound exposure with wild-type running and seizures. By the age of 8 weeks, this percentage of AE-proneness decreased to 40%, with less than 20% of animals displaying seizures [180].

*Vlgr1*-mutated mice represent a “monogenic” model in which the manifestation of AE was highly dependent on genetic background [180]. *Vlgr1*-mutated mice backcrossed with C57BL/6 and 129S1/SvImJ strains were obtained. The 129-backcrossed *Vlgr1*-mutated mice showed a higher incidence of wild running than C57BL/6-backcrossed mice, and C57BL/6-backcrossed *Vlgr1*-mutated mice demonstrated significantly higher mortality after sound exposure than 129-backcrossed mice.

The genetic variant of the *Vlgr1* gene with 14198T > C substitution was also confirmed in the WAR AE model, evidencing the causal connection of this gene with AE proneness [181].

Another gene with Mendelian inheritance associated with multiple central nervous system pathologies including epilepsy in humans is *WWOX* (WW domain-containing oxidoreductase), previously described as a tumor suppressor [182]. Bi-allelic mutations in this gene cause a syndrome called *WWOX-related epileptic encephalopathy* (WOREE) with epilepsy, severe developmental delay, ataxia and premature mortality in childhood [183,184]. To date, the role of the *WWOX* gene in CNS development is not known in detail. A spontaneous *Wwox* mutation in inbred Wistar-Imamichi rats leads to dwarfism, postnatal lethality, male hypogonadism and a high incidence of epilepsy (lethal dwarfism with epilepsy, LDE rats) [185]. Homozygous *Wwox*-mutated rats display hippocampal region vacuolization and AE in 95% of animals. The tissue-specific deletion of the *Wwox* gene in mouse CNS led to a significant decrease in the transcript levels of genes involved in myelination, decreased axon myelination and reduced maturation of oligodendrocytes. There is a known association between myelination disorders and epilepsy [186]. *Wwox*-null mutated mice were predisposed to brain hyperexcitability, intractable epilepsy, ataxia and postnatal lethality [183]. The restoration of *Wwox* expression by neonatal gene therapy using an adenoviral vector carrying *Wwox* cDNA under the control of the synapsin I gene promoter reduced premature mortality and predisposition to AE, as well as promoting partial normalization of the development of *Wwox*-mutated mice [187].

Predisposition to AE and spontaneous death after seizures was shown in serotonin 5-HT2c receptor mutant mice [188]. Audiogenic seizures in this strain started to develop at 60–75 days of age, and by day 120, 100% of animals tested were AE-prone. Data on the role of the serotoninergic system in epilepsy development are inconsistent and depend on the epilepsy form (in humans) or animal model [189,190,191,192,193].

One more gene of interest in terms of inherited causes of epilepsy is *GABRB2*, which encodes the β2-subunit of the gamma-aminobutyric acid receptor—GABA_A_. In humans, various mutant alleles of this gene determine the development of a wide range of epilepsy forms [194,195,196]. A *Gabrb2-/-* knockout mouse strain with a characteristic schizophrenia-like phenotype and AE was obtained [197]. Interestingly, this strain showed signs of neuroinflammation indicated by increased brain levels of the oxidative stress markers, malondialdehyde and proinflammatory cytokines.

The dysfunction of the Lgi1 (leucine-rich repeat glioma-inactivated) protein, originally described as a suppressor of tumor growth and metastasis [198] is associated with epilepsy in humans. Unlike most proteins whose gene mutations are associated with the development of epilepsy, Lgi1 is a secreted protein, the ligand of ADAM22 and ADAM23 (disintegrin and metalloproteinase domain-containing proteins). Lgi1 binds to ADAM22 at the postsynaptic membrane and ADAM23 at the presynaptic membrane, mediating AMPA receptor and potassium channels activity, and thus participating in the regulation of synaptic activity [199,200]. The increased expression of Lgi1 can also inhibit ERK1/2-cascade activity [201]. Finally, Lgi1 regulates the activity of inwardly rectifying K^+^ (Kir) channels, which contain astrocyte Kir4.1 subunits (Kir4.1 channels) involved in the K^+^ homeostasis in the CNS [200]. In humans, at least 43 mutations in the *LGI1* gene are known to be associated with *autosomal dominant lateral temporal lobe epilepsy* (ADLTE) [202]. The autoimmune reaction with the development of autoantibodies to Lgi1 causes a form of autoimmune epilepsy [42,43]. Finally, loss-of-function mutations in the *KCNJ10* gene encoding Kir4.1 cause the epileptic disorders known as “EAST” (*epilepsy, ataxia, sensorineural deafness, and tubulopathy*) [200]. Mutations in the *Lgi1* gene leading to epilepsy cause impaired secretion of the *Lgi1* protein [200,203]. Rats carrying a missense mutation (L385R) in the *Lgi1* gene were obtained. Homozygous *Lgi1*-mutant rats were predisposed to early-onset spontaneous epileptic seizures and died prematurely. Heterozygous *Lgi1*-mutant rats were more susceptible to audiogenic generalized tonic–clonic seizures than wild-type rats. *Lgi1* knockout mice with a similar phenotype were also obtained [204]. The seizure threshold in *Lgi1* heterozygotes decreased with age (13% sensitive animals at 21 days of age, and 52% at 28 days of age) [204].

Recently, a rat strain with a homozygous knockout of the *Kcnj16* encoding K-channel Kir5.1 gene, leading to the development of AE, was obtained as one more model of channalopathy, leading to epilepsy. Mutations in the *KCNJ16* gene were previously found in patients with epilepsy [205,206].

The Black Swiss mouse strain is characterized by partial hearing loss and susceptibility to AE, which reaches a maximum at 2 to 3 weeks of age and then gradually decreases so that by 6 weeks of age, the mice become resistant to loud sounds. A locus within chromosome 10 responsible for the predisposition to AE in Black Swiss mice (juvenile audiogenic monogenic seizure 1, *jams1*) was identified [207]. Originally, two loci designated as age-related hearing loss (*ahl5* at chromosome 10) and *ahl6* (at chromosome 18) were hypothesized to be responsible for hearing loss, and it was believed that the *ahl5* locus made the major contribution to deafness inheritance [208,209]. It was further shown that the *ahl5* and *jams1* loci partially overlap. A mutation in the *Gipc3* (GAIP-interacting protein C terminus) gene, responsible for both progressive hearing loss and juvenile audiogenic epilepsy in Black Swiss mice, was identified [209]. The encoded protein, Gipc3, contains the PDZ domain, which plays a key role in the anchoring of the receptor proteins to the cytoskeletal components. The Gipc3 protein was shown to be co-localized with vesicular glutamate transporter 3 (VGLUT3) and myosin VI, which regulate glutamate release from presynaptic terminals in the inner hair cells, suggesting its possible involvement in excitation transfer by glutamatergic neurons at the level of vesicular transport [209]. Mutations in the *GIPC3* gene are thought to induce *autosomal recessive deafness* (DFNB) but, according to the data available, are not related to human epilepsy [209].

In general, physiological and morphological abnormalities of the auditory system have been described in many rodent strains with AE, but the direct link between hearing impairment and AE has not been confirmed. This suggests that both hearing system abnormalities and AE-proneness could have some common source, i.e., genetic changes leading to abnormal prenatal CNS development [149].

The strain of mice with the *tremor* phenotype was obtained as a result of a spontaneous mutation found in the Swiss-Webster mouse colony (University of São Paulo, Brazil). This strain is characterized not only by audiogenic generalized clonic seizures, but also by body tremor, ataxia, and decreased exploratory behavior. The *tremor* strain is characterized by increased expression (in comparison to wild type) of the *Egr3* (early growth response protein 3) gene, which encodes a transcription factor controlling the expression of GABA_A_ and NMDA receptors [83,210,211]. To date, we have been unable to find data on the association of mutations in this gene with human epilepsy.

A knockout of eukaryotic elongation factor 1Bδ (eEF1Bδ) long isoform encoded by the *Eef1d* gene was obtained from the AE-non-prone C57BL/6J mouse strain. In the knockout mice, the sound-induced seizures were significantly more pronounced and observed in a higher percentage of animals [212]. This factor (eEF1Bδ) is a heat-shock-responsive transcription factor [213]. Mutation of this gene in humans leads to severe intellectual disability and microcephaly [214], whereas no significant developmental or behavioral disorders were identified in knockout mice [212]. Thus, not all genes with mutations leading to AE-proneness are associated with epilepsy in humans.

### 7.3. Models of Pyridoxine-Dependent Epilepsy and Angelman Syndrome

Mouse knockout of three genes from the family, encoding the proline and acidic amino acid-rich basic leucine zipper (PAR bZip) transcription factor, develops spontaneous and audiogenic seizures. The expression of these genes in most tissues obeys circadian rhythmicity, but in brain tissues they are expressed at almost invariable levels. The authors attribute the knockout effect of these transcription factors to the fact that one of their targets is the *Pdxk* gene encoding pyridoxal kinase, which converts vitamin B6 derivatives into pyridoxal phosphate, involved in amino acid and neurotransmitter metabolism. It was further shown that the resulting effect was mainly due to the knockout of one of the three PAR bZip coding genes, *Tef* (thyrotrophic embryonic factor), as the strain with only the *Tef* gene knockout showed a similar convulsive phenotype [215]. The authors believed that this mouse strain could be a model of pyridoxine-dependent human epilepsy. However, in humans, this epilepsy is caused by other gene mutations that are also involved in vitamin B6 metabolism. They are *ALDH7A1* gene (coding alpha-aminoadipic-semialdehyde dehydrogenase) and *PROSC* gene (pyridoxal-5′-phosphate binding protein). Perhaps the mouse model described could be closer to epilepsy caused by a mutation in the gene encoding the pyridoxal-5′-phosphate oxidase (PNPO) responsive to pyridoxal-5-phosphate administration, not to pyridoxine administration [216].

Human Angelman syndrome (AS) manifests as defects in intellectual and speech development, emotional retardation, general motor deficits, and epilepsy in 80% of cases. It is caused by a partial deletion of the 15q11.2-q13.3 region in the maternal chromosome, which leads to either loss or mutation of the *UBE3A* gene encoding the ubiquitin–protein ligase E3A (UBE3A) also known as E6AP ubiquitin–protein ligase (E6AP). This enzyme is involved in targeting proteins for degradation within the proteasome. Inheriting paternal allele mutation in the same locus leads to the development of Prader–Willi syndrome with more mild neurological manifestations. The molecular mechanisms of the pathophysiology and inheritance of this syndrome were previously described [217]. Mutations specifically affecting the *UBE3A* gene occur in 5 to 10% of AS. In more than 75% of cases, large-size deletions within the maternally inherited chromosome 15 appear, herewith the deletion includes not only the *UBE3A* gene, but surrounding sequences containing additional genes (namely *GABRB3*, *GABRA5* and *GABRG3*, encoding different GABA_A_ receptor subunits) [218,219]. To date, several mouse models of AS with a characteristic phenotype have been obtained. The most commonly used model was generated by the deletion of the *Ube3a* exon 5 with a frameshift mutation [220]. Subsequently, several additional *Ube3a* knockout models with different mutations of this gene were obtained (reviewed in [217]). The deletion of the *GABRB3*-*GABRA5*-*GABRG3* gene cluster as a cause of AS was modeled by 1.6 Mb deletion of the region containing *Ube3a*, *Atp10a* and *Gabrb3* genes or by deletion involving the *Ube3a*, *Atp10a*, *Gabrb3*-*Gabra5*-*Gabrg3* cluster (and several other closely located mouse genes). Detailed phenotypic descriptions are not available for all these strains obtained, but all AS mouse models available showed audiogenic seizures which could be treated with AEDs [217,221].

### 7.4. Models with Polygenic or Unknown Inheritance

The first example of a putatively polygenic type of inheritance of AE in rodents is DBA/2J mice. Three loci presumably related to AE in this strain were identified: audiogenic seizure prone 1 (*Asp1*), *Asp2*, and *Asp3* on chromosomes 12, 4, and 7, respectively [26,222]. Subsequently, evidence was obtained that the *Asp1* and *Asp2* loci were mainly responsible for the manifestations of AE [222]. Variations in the coding sequence of the *Kcnj10* gene (localized on chromosome 1) leading to amino acid substitutions in the Kir4.1 potassium channel in DBA/2J relative to the C57BL/6B seizure-insensitive mice were also shown [223]. As cited above, mutations in the *KCNJ10* gene cause epileptic disorders in humans [200]. It is assumed that the functions of the genes localized in the *Asp1* and *Asp2* regions are related to the regulation of Ca^2+^-ATPase activity, which is important for synaptic function, in particular affecting the amount of calcium within the synapse, which, in turn, influences the neurotransmitter release from synaptic vesicles [26,224]. To date, these genes have not been identified. Impaired potassium channel activity and glutamate reuptake, in particular, reduced activity of Kir4.1 in astrocytes of DBA/2J mice in comparison with AE-non-prone C57BL/6, was demonstrated [225]. This observation supports the idea that *Kcnj10* gene polymorphism could play a leading role in DBA/2J-C57BL/6 seizure threshold differences in connection with AE [223].

**Table 1:** Rodent strains—AE models, with description of the confirmed or hypothetical epilepsy-associated genes.

№	Species	Strain	Gene	Protein Function	References
**Monogenic inheritance**					
1	Mouse	*Fmr1* knockout, model of Martin-Bell (fragile X chromosome) syndrome	*Fmr1*	FMRP (fragile X mental retardation protein), mRNA transport	[161,162,163,164]
2	mouse	*Frings*	*Vlgr1*	Very large G-protein coupled receptor 1, cellular adhesion	[22,23,24,165,166,167]
3	rat	LDE, lethal dwarfism with epilepsy rats	*Wwox*	WW domain-containing oxidoreductase	[172]
4	mouse	CNS-specific *Wwox* knockout mice	*Wwox*	WW domain-containing oxidoreductase	[173]
5	mouse	5-HT2c receptor mutant mice	*Htr2c*	Serotonin receptor	[175]
6	mouse	*Gabrb2*-/- knockout	*Gabrb2*	β2-subunit of the gamma-aminobutyric acid receptor	[184]
7	rat	*Lgi1*-mutant rats	*Lgi1*	Secreted protein, modulator of disintegrin and metalloproteinase domain-containing proteins and K-channel activity	[191]
8	mouse	*Lgi1*-mutant mice	*Lgi1*	Secreted protein, modulator of disintegrin and metalloproteinase domain-containing proteins and K-channel activity	[191]
9	rat	*Kcnj16*-knockout	*Kcnj16*	Kir5.1 potassium channel	[192,193]
10	mouse	*Black Swiss* *	*Gipc3*	GAIP-interacting protein C terminus, anchoring of the receptor proteins to the cytoskeleton	[194,195,196]
11	mouse	*Tremor*	*Egr3*	Early growth response protein 3, transcription factor	[197]
12	mouse	Knockout of eukaryotic elongation factor 1Bδ (eEF1Bδ) long isoform	*Eef1d*	Eukaryotic elongation factor 1Bδ (eEF1Bδ) long isoform	[199]
13	mouse	*Tef* knockout mouse strain	*Tef*	Proline and acidic amino acid-rich basic leucine zipper (PAR bZip) transcription factor	[202]
14	mouse	*Ube3a* mutated mice, model of Angelman syndrome	*Ube3a*	Ubiquitin–protein ligase E3A	[204,207]
**Polygenic or putatively polygenic inheritance**					
15	mouse	DBA/2J	*Kcnj10*	Kir4.1 potassium channel	[209,210]
16	mouse	101/HY	unknown	Unknown	[213]
17	rat	GEPR, genetically epilepsy-prone rats	unknown	Unknown	[137,214,215]
18	rat	WAR, Wistar audiogenic rats	Hypothetically: *Vlgr1* *Chrna4* *Grin2a* *Grin2b* *Kcnq3* *Egr3* *Ttr*	Very large G-protein coupled receptor 1, cellular adhesion Nicotinic acetylcholine receptor Glutamate (NMDA) receptor subunit Glutamate (NMDA) receptor subunit Voltage-gated potassium channel Early growth response protein, transcription factor Transthyretin, transport protein	[83,168,198,217,218,222]
19	rat	KM, Krushinsky-Molodkina	Hypothetically: *Ttr* *Msh3* *Gstm1*	Transthyretin, transport protein MutS Homolog 3, DNA mismatch repair Glutathione S-transferase Mu 1, sulfur metabolism, detoxification	[115]
20	hamster	GASH/Sal, genetic audiogenic seizure hamster, Salamanca	Hypothetically: *Cacna1a* *Cacna2d3* *Grik1* *Grin2c* *Zeb2* *Egr3* *Ttr* *Msh3*	Calcium voltage-gated channel subunit Calcium voltage-gated channel subunit Glutamate ionotropic receptor kainate type subunit 1 Glutamate (NMDA) receptor subunit ε-3 Zinc finger E-box-binding homeobox 2, transcription factor Early growth response protein, transcription factor Transthyretin, transport protein MutS Homolog 3, DNA mismatch repair	[217,219,221]
21	mouse	*Ube3a*-deleted mice, model of Angelman syndrome	*Ube3a* *Atp10a* *Gabrb3*	Ubiquitin-protein ligase E3A Phospholipid-transporting ATPase VA (aminophospholipid translocase VA) β3-subunit of the gamma-aminobutyric acid receptor	[204]
22	mouse	Del(7*Gabrb3*-*Ube3A*), model of Angelman syndrome	*Ube3a* *Atp10a* *Gabrb3* *Gabra5* *Gabrg3* *Oca2* *Herc2*	Ubiquitin-protein ligase E3A Phospholipid-transporting ATPase VA (aminophospholipid translocase VA) β3-subunit of the gamma-aminobutyric acid receptor α5-subunit of the gamma-aminobutyric acid receptor γ3-subunit of the gamma-aminobutyric acid receptor Melanocyte-specific transporter protein, membrane transport Giant E3 ubiquitin protein ligase	[204]

* Was previously considered as a strain with polygenic inheritance.

There are other strains of mice with presumably polygenic inheritance of AE, such as 101/HY [226], but it is unknown which mutations are responsible for AE-proneness in these strains.

Two substrains were selected for AE based on the Sprague Dowely population: GEPR-3, displaying moderate seizure intensity, and GEPR-9 with severe AE seizures [26]. Sound-induced seizures appear in GEPR-9 at the age of 25–35 days, with a further increase in intensity and decrease in fit latency [227]. Genetic studies indicate a polygenic inheritance of AE-proneness in GEPRs, while genes responsible for AE are still unknown [143,228]. At the same time, GEPRs’ GABAergic system anomalies were described in detail [26,229].

The genetics of AE development in three rodent strains obtained by classical selection with a presumably polygenic inheritance of this trait, WAR, KM and GASH/Sal, were described in detail. In KM rats, the audiogenic phenotype appears at 1 month of age, and by the age of 3 months, 100% of animals are AE-prone [25]. In the GASH/Sal strain, the age of AE expression start was not indicated. In WAR, the stable AE phenotype is present at the age of 70–78 days [25]. The cDNA microarray and RNA-seq methods identified the genetic abnormalities in connection with AE-proneness in these strains, followed by mutations in exon sequences, determining the expression of a wide range of genes and differences in the activity of key signaling pathways, demonstrated by the comparison of AE-prone and control strains [121,230,231,232,233]. In WAR and GASH/Sal, the transcriptome of the IC, the structure, determining the start of AE seizure, was analyzed for both groups—animals not exposed to sound (naïve) and for animals after a seizure [230,234]. In KM rats, the transcriptome of the IC and SC of naïve animals was analyzed [121]. It is the profile of the transcriptome in naïve animals that is of maximal interest, as it allows to evaluate the contribution of various genes and signaling pathways to the AE-proneness. On the other hand, changes in the genes expression levels after seizures may include those involved in the compensatory mechanisms of seizure consequences [121]. However, the identification of specific genes with mutations and/or changes in expression levels that lead to seizure development in epilepsy models with polygenic or unknown nature of inheritance using whole-genome analysis methods faces a fundamental issue which complicates the whole problem. The thing is that most “classical” AE-prone strains (KM, GEPRs, WAR, and GASH/Sal) were bred by selection several decades ago, and after this, they were maintained in isolation from the original populations for a long time. This isolated breeding makes the direct comparison between AE-prone and AE-non-prone strains not very informative, as many genetic events could occur (both in initial and selected strains). The majority of such events should be neutral, i.e., not associated with the trait investigated—AE-proneness. Thus, 71 differential-expressing genes (DEGs) for the WAR model and 64 DEGs for GASH/Sal (in AE vs. non-AE comparison) were detected [130]. A comparison of the transcriptomes of KM and Wistar rats revealed 1488 DEGs [121]. Finding the genes responsible for AE in this dataset is a challenging goal. A plausible method of analysis is the search for orthologous genes associated with human epilepsy among the DEGs found in AE-prone rat strains. However, this may not be sufficient, as not all genes causing human epilepsy are discovered. Moreover, several mutations were described that have different effects in rodents and humans. For instance, mutations in the *Eef1d* gene (see above) do not influence development and behavior, but cause AE in mice, while in humans, similar mutations induce severe intellectual disability and microcephaly [212].

The search for genetic and biochemical peculiarities common to various AE-prone strains resulting from spontaneous mutations with further selection could be informative for revealing mechanisms of AE. Since, as mentioned above, some predisposition to AE is characteristic of rodents in general, we can assume the existence of some genetic pattern leading to this phenotype common to different strains and species. In this regard, it is of interest to compare different models for which RNA-seq data are available, i.e., WAR, KM, and GASH/Sal. Analysis of the IC transcriptome of the GASH/Sal hamster and WAR strains revealed several DEGs common to these strains in comparison to the original AE-non-prone strains—this list of genes includes *Egr3*, *Rgs2*, *Ttr* and *Npy* in both AE models [230,234]. A study of the IC and SC transcriptome in KM rats showed the significantly increased expression of the *Ttr* gene compared with that in naïve Wistar rats. This trait appears to be common for three genotypes—GASH/Sal, WAR and KM [121,230,234]. In addition, the expression of the *Msh3* gene was significantly (5 to 10-fold) reduced in KM rats compared with the Wistar strain. Another list of DEGs for KM and Wistar pair did not overlap with those found for WAR and GASH/Sal. Thus, KM rats (compared with Wistar) demonstrated a decrease in the expression of several mitochondrial enzymes genes (in particular, *Acsm5* encoding acyl-CoA synthetase medium-chain family member 5), several respiratory chain components, the *Cacng4* gene (Voltage-dependent calcium channel gamma-4 subunit), *Igfbp5* (insulin-like growth factor-binding protein 5) and *Gstm1* (glutathione S-transferase Mu 1) genes. The involvement of most of the DEGs detected in the epileptogenesis has not been proven yet.

The *Egr3* gene overexpressed in WAR and GASH/Sal encodes a transcriptional regulator that belongs to the EGR (early growth response proteins) family of C2H2-type zinc-finger proteins and regulates NMDA receptor 1 and GABA_A_ receptor α4 subunit via the BDNF-PKC/MAPK signaling pathway [83,211,235]. Increased expression of GABA_A_ receptor α4 subunit and, conversely, decreased expression of α1 subunit of this receptor plays an important role in human *temporal lobe epilepsy* and in a mouse model of pilocarpine-induced seizures [83,235,236]. Thus, increased expression levels of *Egr3* may play a causative role in the development of AE in WAR and GASH/Sal as well. The AE-prone *tremor* mouse strain is also characterized by increased expression of the *Egr3* gene [210]. On the other hand, we were unable to find data on the association of the *Egr3* gene with human epilepsy. Mutations in the *Ttr* gene in humans cause family amyloid polyneuropathy but not epilepsy [237,238]. It is the only gene for which overexpression was found in WAR, KM and GASH/Sal, compared with the original AE-non-prone strains [121]. Transthyretin encoded by the *Ttr* gene regulates the activity of GABA_A_ receptors, which play an important role in the control of predisposition to seizures [239]. Deficiencies of GABA_A_ receptor-mediated neurotransmission were previously shown in GASH/Sal hamster and WAR rat models. In KM rats, the imbalance of glutamate-GABA content was noted at a neurochemical level as well [66,142,230,232]. On the other hand, several studies reported the neuroprotective role of transthyretin in Alzheimer’s disease and in cases of cobalt-induced seizures in animals [240,241,242]. Therefore, overexpression of the *Ttr* gene in AE-prone models may be regarded as a compensatory mechanism. The *Rgs2* gene encodes a protein that modulates G protein-coupled receptor signaling cascades (GPCRs), the so-called G protein signaling regulator 2 (RGS2) [243]. An increase in this gene expression occurred after electroconvulsive stimulation, and this change could be also of adaptive significance [244]. In humans, the decreased expression of the *Rgs2* gene is associated with mild cognitive impairment and several neurodegenerative diseases [245]. Neuropeptide Y, encoded by the *Npy* gene, is characterized by anticonvulsant function. Its increase in WAR and GASH/Sal in comparison with AE-non-prone animals also looks like an adaptive change [40,246]. Among the DEGs found in KM and Wistar transcriptomes compared, the association with human epilepsy was found for the orthologue of the *Gstm1* gene only [121,247,248].

In addition to DEGs, sequencing of the transcriptome of GASH/Sal animals in comparison with the control AE-non-prone strain revealed several mutations in genes’ coding regions, and several of these genes were described as being associated with human epilepsy. In this list, mutations in the *Cacna1a* and *Cacna2d3* genes encoding subunits of the calcium voltage-gated channel are of the most interest [232]. In humans, mutations in the *CACNA1A* induced *developmental epileptic encephalopathies* (DEEs) [249]. *Grik1* (glutamate ionotropic receptor kainate type subunit 1) gene polymorphism was found in hamsters, while the human ortholog is associated with epilepsy development, including *juvenile absence epilepsy* (JAE) [232,250]. *Cacna1a* and *Grick1*, together with *Grin2c*, in which a substitution was also found in GASH/Sal, are parts of the glutamatergic synapse pathway, which is enhanced in GASH/Sal [232]. The *Grin2c* gene encodes the glutamate (NMDA) receptor subunit ε-3 [251]. Mutations in genes encoding glutamate receptor subunits ε-1 (*GRIN2A*) [252] and ε-2, i.e., genes related to previously mentioned (*GRIN2B*) [253], are also associated with human epilepsy. One more GASH/Sal gene *Zeb2* (encoding Zinc finger E-box-binding homeobox 2) carries the substitution (in comparison to the control strain) and is possibly associated with seizure development. The encoded protein is the transcription factor, which participates in the transforming growth factor β (TGFβ) signaling pathway and is essential for early fetal development. Mutations in the *ZEB2* gene in humans are associated with Mowat–Wilson syndrome, a complex disease with epilepsy and severe CNS developmental abnormalities [232,254,255]. Mutations in several other genes found in GASH/Sal are probably neutral.

WAR transcriptome analysis revealed mutations in *Chrna4*, *Grin2a*, *Grin2b*, *Kcnq3*, *Vlgr1* and several other genes [181]. *Chrna4* encodes a subunit of the nicotinic acetylcholine receptor, and its mutations are associated with *nocturnal frontal lobe epilepsy*. *Kcnq3* encodes a subunit of the voltage-gated potassium channel with mutations associated with *benign familial neonatal seizures* [256]. Mutations in the *Vlgr1* gene are associated with human epilepsy and with AE in the Frings mice (see above) [181]. Thus, mutations found in GASH/Sal and WAR and absent in AE-non-prone strains affect mainly voltage-gated ion channels and glutamatergic system. In addition, several thousands of apparently neutral polymorphisms between WAR and Wistar strains were detected using RNA-seq [181].

One should note that no mutations in exon regions of any genes, associated with the development of epilepsy in humans, were found in the KM rat transcriptome analysis [121].

At the same time, GASH/Sal mutations in the *Msh3* and *Ttr* genes were found in addition to those listed above. The *Msh3* gene encodes a homolog of the bacterial protein mutS, which forms a heterodimer with the Msh2 protein responsible for DNA mismatch repair [257]. In the case of the *Msh3* gene, one of the two mutations found in the GASH/Sal strain apparently prevents the interaction of the *Msh3* protein with *Msh2* [232]. No association with epilepsy in humans had been described for the *MSH3* and *TTR* genes, although human *TTR* gene mutations cause family amyloid polyneuropathy [237,238]. The level of the *Msh3* gene expression is significantly higher in GASH/Sal (in comparison with the control strain), and those plausibly could be a compensatory mechanism for the inactivity of the mutant *Msh3* protein [232].

No mutations in the coding region of the *Msh3* gene were found in KM rats, but a significant decrease in the transcription level of this gene (in comparison to Wistars) was noted [121]. It has previously been shown that decreased levels of the Msh3 partner protein, Msh2, in mice lead to increased susceptibility to kainic acid (KA)-induced seizures due to mitochondrial dysfunction [258]. Signs of mitochondriopathy in the form of reduced ATP production and increased H_2_O_2_ levels in brain and liver tissues compared with Wistar rats were also found in KM rats [122]. As mentioned above, transcriptomic analysis shows a decrease in *Acsm5* gene expression, as well as in several genes encoding components of the respiratory complexes and F_0_-ATP synthase in KM rats, which could indicate the mitochondriopathy in these animals [121]. In humans, no association of *Msh3* gene mutations with epilepsy has been found, although mitochondriopathies (induced by mutations in several genes encoding mitochondrial proteins) are known to be associated with epilepsy in 40% of cases [259]. Thus, a leading role of abnormalities in the function or in the expression level of the *Msh3* gene in rodent AE development seems probable. In addition, disorders of the DNA repair system may increase the general frequency of mutations and thus facilitate the selection for the AE phenotype. Interestingly, the 101/HY mouse strain which carries a mutation in the gene locus controlling DNA repair after chemical mutagenesis was found to be AE-prone [226].

The comparison of signaling pathways, involved in synaptic regulation mechanisms, could reveal the common pattern of deviations that are specific to the ”epileptic brain” both in AE models and in humans. This pattern could be found despite the differences described in the mutation “list” for AE strains and for that associated with human epilepsy. From this point of view, the KM rat strain [121] was investigated in more detail. In the KM rats’ brain, increased activity of the MAPK signaling cascade was found which has direct pro-epileptogenic effects in the corpora quadrigemina, as well as proapoptotic and proinflammatory signaling pathways differences from Wistar [121]. MAPK/ERK1/2 activity presumably contributes to seizure threshold decrease via the upregulation of glutamatergic synaptic transmission and suppression of GABA_A_ receptors functions [53,54,55,56,64,260,261]. It is interesting to note that the cDNA expression array performed using the Chinese AE-prone P77PMC rat strain showed a significant increase in ERK2 expression in the cerebral cortex relative to the original AE-non-prone strain [145].

As already mentioned above, in the selection and maintenance of rodent AE strains (and control non-AE strains as well), neutral as well as AE-provoking mutations could be accumulated in the genomes. This makes the correct comparison between AE and control animals a rather problematic one. To facilitate the search for genes associated with AE, the new strain was selected based on F2 (KM x Wistar) hybrids with two back-crosses to the KM strain. As the result, the strain obtained is phenotypically intermediate (in respect to AE phenotype expression) between KM (100% AE-prone) and Wistar (10–15% AE-prone). This strain was named “0”, with 30–60% of AE-prone animals with a fit intensity of a low level, with only AE-non-prone animals being used in the study [121,142,262,263]. Transcriptome sequencing in animals of this “0” strain shows a gene expression profile also intermediate between KMs and Wistars. In AE-non-prone animals of this strain, the expression level of *Msh3*, *Ttr*, *Ascm5*, *Gstm1* and several other genes is close to the norm, i.e., to the expression level close to that in AE-non-prone Wistar rats. The activity of MAPK/ERK1/2 cascade and proapoptotic signaling pathways [121] is also normalized in the “0” animals in comparison to KM. Thus, the data available suggest the participation of these genes and signaling pathways in AE development.

Summarizing the data displayed above, one may conclude that the participation of certain genes in polygenic AE strains could be confirmed by their direct knockouts only, thus confirming (or rejecting) the respective hypothesis.

## 8. The Use of Rodent Strains with AE in AEDs Screening

All AEDs currently used in clinics are characterized by a wide range of side effects, including rather severe ones, and they are not effective in all cases of epilepsy. For this reason, the search for new AEDs continues in order to find drugs with high efficacy and a smaller spectrum of side effects than those currently used. The development of new AEDs mainly includes studies of modified analogs of conventional drugs, possessing fewer side effects [264]. In addition, research continues on the mechanisms of epileptic seizures and on the search for new target proteins, such as cholesterol metabolism enzymes [265].

As mentioned above, the “gold standard” models in AEDs testing are the maximal electroshock technique and the PTZ-induced seizure model [266,267,268]. At the same time, the use of PTZ models proved to be sometimes ineffective, e.g., the absence of the anticonvulsant effect of levetiracetam using this type of seizure [269], whereas in both AE and other types of seizure induction, this drug was effective [270,271]. Numerous investigations proved that clinically approved AEDs reduce effectively the seizure intensity in AE-prone rodents. Numerous data, obtained in this field, confirm the applicability of AE-prone rodents for new AEDs generation testing [135,272,273].

In general, there are probably too many AE-prone rodent strains available today for screening new anticonvulsants and for analyzing the mechanisms of their efficiency. Actually, several strains have rather explicit neurophysiological and biochemical characteristics to be used for this purpose. They are DBA/2 mice, WAR, GEPRs, KM rats and GASH/Sal. Appropriate genetic models described earlier are now used to develop specific treatments for some definite human pathologies, such as fragile X syndrome, Angelman syndrome, etc. [177,274,275].

The DBA/2 mouse strain was used to reveal the anticonvulsant effects of clobazam [276]; benzodiazepine drugs [277]; adenosine (Ado) type 1 receptors (A1Rs) and their ligands [278,279]; effects of serotoninergic system enhancing substances [280]; and structurally diverse GABA_B_ positive allosteric modulators [281]. The DBA/2 strain was also used to study the synergism of carbenoxolone (the succinyl ester of glycyrrhetinic acid, an inhibitor of 11beta-hydroxy steroid dehydrogenase) and conventional antiepileptic drugs (carbamazepine, diazepam, felbamate, gabapentin, lamotrigine, phenytoin, phenobarbital and valproate) [282] as well as the pharmacodynamic potentiation of antiepileptic drugs’ effects by HMG-CoA reductase inhibitors [283]. In Frings mice, the effects of a new analog of topiramate [264], the anticonvulsive properties of soticlestat, a novel cholesterol 24-hydroxylase inhibitor [265], and the effects of synaptic and extrasynaptic GABA transporters inhibitors were investigated [284]. *Fmr-/-* mice were used to analyze the possibility of mGluRs and GABA receptors’ activity modulation in fragile X syndrome therapy [177,275]. The anticonvulsant properties of lovastatin were investigated in fragile X and Angelman syndrome models [274]. In GEPR-3 and GEPR-9 strains, the anticonvulsant effect of several ion-exchange transporter inhibitors [285] and the ability of liraglutide to reduce tolerance to diazepam [286] were studied. The KM strain was used in studies of the ability of vigabatrin (a GABA decay inhibitor) to reduce the clonic component of audiogenic seizures [287]. Additionally, KM rats were used in studies of the antiepileptic potential of ERK1/2 inhibitors [59,64]. In WAR, the anticonvulsant effects of carisbamate, a novel neuromodulator for the treatment of epilepsy [288], nifedipine (calcium channel inhibitor) [289] and cannabidiol [290] were studied. Finally, pharmacological and neuroethological studies of the acute and chronic effects of lamotrigine, phenobarbital and valproic acid were performed using the GASH/Sal model [291,292].

In general, one may conclude that along with standard models of seizures induced by maximal electroshock and PTZ, the AE-prone rodents could be widely used in the testing of old and new AEDs.

## 9. Near-Term Prospects and Conclusions

There is currently a rapidly expanding set of new epilepsy models with monogenic inheritance obtained by directed mutagenesis, due to the amazing success of genetic engineering. These achievements have promoted an understanding of the molecular mechanisms of epileptogenesis. In monogenic models, it is possible to establish an unambiguous causal relationship between a known mutation and the development of epileptic events. One may assume that large collections of knockout and transgenic mice (such as Jackson Laboratory), may possess several mutant strains that are predisposed to AE, but this trait has not yet been revealed. This assumption seems legitimate, given that in rodents the AE trait (in some cases) could be determined by mutations in genes for which no association with human epilepsy has been discovered [215,293]. An example of such incidental finding of gene knockout association with a predisposition to AE was mentioned previously [215]. Interestingly, the lethal audiogenic seizures in mice with knockout of three PAR bZip genes occurred on definite days of the week. Subsequently, it turned out that the staff was cleaning the lab using a noisy vacuum cleaner on these particular days. Thus, some unobvious relationships between genes with a not-described association with epilepsy and the development of AE could be found by testing for AE of a wide set of mutant rodent strains available.

The available set of rodent AE-prone strains with a monogenic type of inheritance demonstrates a significant diversity of genes involved in various regulatory processes. These are mutations of genes that control the expression of AE: transcription factors (*Egr3*, *Tef*), enzymes (*Wwox*), receptors (*5-HT2c*, *Vlgr1*, *Gabrb2*), anchor proteins (*Gipc3*), regulators of mRNA translation and transport (*Fmr1*), etc.

Thus, the models of various forms of epilepsy are available for epilepsy syndromes with different etiologies and in which definite CNS mechanisms are involved, including action potential generation and early developmental events. In several AE models, the altered function of signaling cascades was also described (e.g., MAPK in KM rats) [121] as well as the development of neuroinflammation [197]. Thus, the AE models available provide an opportunity to test a wide range of potential AEDs targeting different proteins involved in various cellular processes, the disruption of which could induce epileptogenesis. Moreover, knockout mouse strains represent an obligatory model system for the development of epilepsy gene therapy methods based on CRISPR/Cas or adenoviral systems targeting, i.e., the development of the individual (associated with epilepsy) mutations “corrections” [293].

Rodent AE models with polygenic inheritance (KM, GEPRs, WAR and GASH/Sal) were developed as the result of numerous spontaneous mutations’ accumulation. Only part of these mutations is responsible for AE-proneness, while others are neutral or even exert compensatory effects for the AE phenotype (having been involuntarily selected for during selection generations). On the one hand, the identification of the genes responsible for audiogenic seizures in these models faces rather serious problems. It is known that several mouse knockouts of genes, whose human orthologs are associated with epilepsy, do not display neither AE nor spontaneous seizures in mice [293]. As an example, *Tsc1* and *Tsc2* (Tuberous sclerosis complex) knockouts do not lead to the development of seizures in mice, unlike tuberous sclerosis cases in humans [293,294]. On the other hand, the opposite is also true, i.e., several mutations were described that induce seizures in rodents, and definite disorders in humans [83,210,211]. Pathological deviations that lead to epileptogenesis in AE-prone strains obtained by selection in independent experiments obviously do not coincide with one another because they could be determined by different mutations. Nevertheless, they could lead to similar results, demonstrating violations of glutamate and GABAergic systems, which provoke seizures. Polygenic AE animal models could be indispensable for studying human epilepsy with a complex type of inheritance. The role of genetic pathology patterns when mutations of different alleles of multiple genes combine with genetic background characteristics (which is observed, in particular, in the KM model), should be analyzed in more details to understand the nature of many complicated forms of epilepsy [9,295,296].

Most monogenic mouse models of epilepsy were obtained by introducing mutations into the coding regions of target genes (reading frame shifts or substitutions). Many epilepsy cases in humans also describe mutations in the coding regions of certain genes, leading to the loss of functions of the protein encoded. At the same time, a lot of human epilepsy cases were described with mutations affecting not the coding, but the regulatory region of genes [297,298]. In such cases, functional disturbances occur due to changes in expression levels relative to the normal ones. In this aspect, the KM rat strain is indicated (in comparison to other rodent models), in which no mutations were found in candidate genes for association with epilepsy, but a wide range of candidate genes could be indicated in which the expression is significantly increased or decreased relative to the conditional norm of Wistar rats [121].

Gene expression could be also affected by the methylation status of gene promoters, as was demonstrated for epilepsy-associated genes *FMR1* and *BRD2* (bromodomain-containing transcriptional activator 2) in humans [172,299]. The expression level of many genes could also be regulated by the RNA interference mechanism involving miRNAs (small non-coding RNAs that regulate post-transcriptional gene expression) [300]. It was found that in some cases, miRNAs as well as long non-coding RNAs can act as biomarkers and therapeutic targets in human epilepsy [301,302,303,304,305,306]. Thus, in the case of some rodent models with polygenic inheritance obtained by the selection, the search for mutations in the regulatory regions of some genes, for epigenetic changes, and changes in the expression of interfering RNAs represent a mandatory field for future research.

Transcriptomic studies in WAR, KM strains and GASH/Sal were carried out using adult animals 3 months of age, after the full development of the AE phenotype [121,230]. At the same time, it is also important to analyze CNS disorders during pre- and postnatal development in these animals, which could lead to subsequent AE development [149]. It is plausible that the important changes in gene expression, leading to future epileptic events emergence, could occur at the early stages of ontogeny which should also be investigated by RNA-seq methods in the future.

## Abbreviations

AE	audiogenic epilepsy
AEDs	antiepileptic drugs
AMPA	α-amino-3-hydroxy-5-methyl-4-isoxazolepropionic acid
AS	Angelman syndrome
CAE	childhood absence epilepsy
CNS	central nervous system
DEEs	developmental epileptic encephalopathies
DEGs	differential-expressing genes
EAST	epilepsy, ataxia, sensorineural deafness, and tubulopathy
EEG	electroencephalography
EIEE	early infantile epileptic encephalopathy
ERK	extracellular signal-regulated kinase
GABA	gamma-aminobutyric acid
GASH/Sal	genetic audiogenic seizure hamster from Salamanca
GEPR	genetically epilepsy-prone rat
IC	inferior colliculi
ILAE	The International League Against Epilepsy
JAE	including juvenile absence epilepsy
JME	juvenile myoclonic epilepsy
KM	Krushinsky–Molodkina
MAG	myelin-associated glycoprotein
MAPK	mitogen-activated protein kinase
MES	maximal electroshock test
Msh3	MutS Homolog 3
mtDNA	mitochondrial DNA
mTOR	mammalian target of rapamycin
NMDA receptors	N-methyl-D-aspartate receptor
PAR bZip	proline and acidic amino acid-rich basic leucine zipper
PTZ	pentylentetrazole
SC	superior colliculi
TGFβ	transforming growth factor β
tRNA	transfer RNA
Ttr	transthyretin
WAR	Wistar audiogenic rat
WHO	World Health Organization
WOREE	WWOX-related epileptic encephalopathy
WWOX	WW-domain-containing oxidoreductase

## References

[1] Betjemann J.P., Lowenstein D.H. (2015). Status epilepticus in adults. Lancet Neurol..

[2] Zimmern V., Korff C. (2020). Status Epilepticus in Children. J. Clin. Neurophysiol..

[3] Bolton P.F., Carcani-Rathwell I., Hutton J., Goode S., Howlin P., Rutter M. (2011). Epilepsy in autism: Features and correlates. Br. J. Psychiatry.

[4] Strasser L., Downes M., Kung J., Cross J.H., De Haan M. (2018). Prevalence and risk factors for autism spectrum disorder in epilepsy: A systematic review and meta-analysis. Dev. Med. Child Neurol..

[5] Alsaadi T., El Hammasi K., Shahrour T.M., Shakra M., Turkawi L., Almaskari B., Diab L., Raoof M. (2015). Prevalence of depression and anxiety among patients with epilepsy attending the epilepsy clinic at Sheikh Khalifa Medical City, UAE: A cross-sectional study. Epilepsy Behav..

[6] Josephson C.B., Jetté N. (2017). Psychiatric comorbidities in epilepsy. Int. Rev. Psychiatry.

[7] Hassani M., Cooray G., Sveinsson O., Cooray C. (2020). Post-stroke epilepsy in an ischemic stroke cohort-Incidence and diagnosis. Acta Neurol. Scand..

[8] Fordington S., Manford M. (2020). A review of seizures and epilepsy following traumatic brain injury. J. Neurol..

[9] Wang J., Lin Z.J., Liu L., Xu H.Q., Shi Y.W., Yi Y.H., He N., Liao W.P. (2017). Epilepsy-associated genes. Seizure.

[10] Perucca P., Bahlo M., Berkovic S.F. (2020). The Genetics of Epilepsy. Annu. Rev. Genom. Hum. Genet..

[11] Löscher W., Klitgaard H., Twyman R.E., Schmidt D. (2013). New avenues for anti-epileptic drug discovery and development. Nat. Rev. Drug Discov..

[12] Vermeulen J., Aldenkamp A.P. (1995). Cognitive side-effects of chronic antiepileptic drug treatment: A review of 25 years of research. Epilepsy Res..

[13] Chen B., Choi H., Hirsch L.J., Katz A., Legge A., Buchsbaum R., Detyniecki K. (2017). Psychiatric and behavioral side effects of antiepileptic drugs in adults with epilepsy. Epilepsy Behav..

[14] Carreno M. (2007). Levetiracetam. Drugs Today.

[15] Zhang L.L., Zeng L.N., Li Y.P. (2011). Side effects of phenobarbital in epilepsy: A systematic review. Epileptic Disord..

[16] Shimada T., Yamagata K. (2018). Pentylenetetrazole-Induced Kindling Mouse Model. J. Vis. Exp..

[17] Williamson J., Singh T., Kapur J. (2019). Neurobiology of organophosphate-induced seizures. Epilepsy Behav..

[18] Patra P.H., Barker-Haliski M., White H.S., Whalley B.J., Glyn S., Sandhu H., Jones N., Bazelot M., Williams C.M., McNeish A.J. (2019). Cannabidiol reduces seizures and associated behavioral comorbidities in a range of animal seizure and epilepsy models. Epilepsia.

[19] Banach M., Borowicz K.K. (2015). Effects of Chronic Lamotrigine Administration on Maximal Electroshock- Induced Seizures in Mice. CNS Neurol. Disord. Drug Targets.

[20] Gonzalez D., Tomasek M., Hays S., Sridhar V., Ammanuel S., Chang C.W., Pawlowski K., Huber K.M., Gibson J.R. (2019). Audiogenic Seizures in the. J. NeuroSci..

[21] Ross K.C., Coleman J.R. (2000). Developmental and genetic audiogenic seizure models: Behavior and biological substrates. NeuroSci. Biobehav. Rev..

[22] Skradski S.L., Clark A.M., Jiang H., White H.S., Fu Y.H., Ptácek L.J. (2001). A novel gene causing a mendelian audiogenic mouse epilepsy. Neuron.

[23] Yagi H., Takamura Y., Yoneda T., Konno D., Akagi Y., Yoshida K., Sato M. (2005). Vlgr1 knockout mice show audiogenic seizure susceptibility. J. Neurochem..

[24] Myers K.A., Nasioulas S., Boys A., McMahon J.M., Slater H., Lockhart P., Sart D.D., Scheffer I.E. (2018). ADGRV1 is implicated in myoclonic epilepsy. Epilepsia.

[25] Doretto M.C., Fonseca C.G., Lôbo R.B., Terra V.C., Oliveira J.A., Garcia-Cairasco N. (2003). Quantitative study of the response to genetic selection of the Wistar audiogenic rat strain (WAR). Behav. Genet..

[26] Italiano D., Striano P., Russo E., Leo A., Spina E., Zara F., Striano S., Gambardella A., Labate A., Gasparini S. (2016). Genetics of reflex seizures and epilepsies in humans and animals. Epilepsy Res..

[27] Berg A.T., Berkovic S.F., Brodie M.J., Buchhalter J., Cross J.H., van Emde Boas W., Engel J., French J., Glauser T.A., Mathern G.W. (2010). Revised terminology and concepts for organization of seizures and epilepsies: Report of the ILAE Commission on Classification and Terminology, 2005–2009. Epilepsia.

[28] Porter R.J. (1993). The absence epilepsies. Epilepsia.

[29] Spencer S.S. (2002). Neural networks in human epilepsy: Evidence of and implications for treatment. Epilepsia.

[30] Vinogradova L.V. (2010). Interhemispheric difference in susceptibility to epileptogenesis: Evidence from the audiogenic kindling model in Wistar rats. Brain Res..

[31] Connolly M.B. (2016). Dravet Syndrome: Diagnosis and Long-Term Course. Can. J. Neurol. Sci..

[32] Keezer M.R., Sisodiya S.M., Sander J.W. (2016). Comorbidities of epilepsy: Current concepts and future perspectives. Lancet Neurol..

[33] Okudan Z.V., Özkara Ç. (2018). Reflex epilepsy: Triggers and management strategies. Neuropsychiatr. Dis. Treat..

[34] Nolan D., Fink J. (2018). Genetics of epilepsy. Handb. Clin. Neurol..

[35] Oyrer J., Maljevic S., Scheffer I.E., Berkovic S.F., Petrou S., Reid C.A. (2018). Ion Channels in Genetic Epilepsy: From Genes and Mechanisms to Disease-Targeted Therapies. Pharmacol. Rev..

[36] Lin Lin Lee V., Kar Meng Choo B., Chung Y.S., P Kundap U., Kumari Y., Shaikh M.F. (2018). Treatment, Therapy and Management of Metabolic Epilepsy: A Systematic Review. Int. J. Mol. Sci..

[37] Reddy C., Saini A.G. (2021). Metabolic Epilepsy. Indian J. Pediatr..

[38] Alizadeh Khatir A., Moghaddam S.A., Almukhtar M., Ghorbani H., Babazadeh A., Mehravar S., Rostami A. (2021). Toxoplasma infection and risk of epilepsy: A case-control study of incident patients. Microb. Pathog..

[39] Nikbakht F., Mohammadkhanizadeh A., Mohammadi E. (2020). How does the COVID-19 cause seizure and epilepsy in patients? The potential mechanisms. Mult. Scler. Relat. Disord..

[40] Cattaneo D., Giacomelli A., MiniSci D., Astuti N., Meraviglia P., Gervasoni C. (2020). Association of HIV Infection with Epilepsy and Other Comorbid Conditions. AIDS Behav..

[41] Gripper L.B., Welburn S.C. (2017). The causal relationship between neurocysticercosis infection and the development of epilepsy—A systematic review. Infect. Dis. Poverty.

[42] Husari K.S., Dubey D. (2019). Autoimmune Epilepsy. Neurotherapeutics.

[43] Geis C., Planagumà J., Carreño M., Graus F., Dalmau J. (2019). Autoimmune seizures and epilepsy. J. Clin. Investig..

[44] Spatola M., Dalmau J. (2017). Seizures and risk of epilepsy in autoimmune and other inflammatory encephalitis. Curr. Opin. Neurol..

[45] Younas A., Saim M., Maqsood H., Younus S., Hassan Raza M. (2020). Dyke-Davidoff-Masson Syndrome: A Case Report and Review of Literature. Cureus.

[46] Specchio N., Pietrafusa N. (2020). New-onset refractory status epilepticus and febrile infection-related epilepsy syndrome. Dev. Med. Child Neurol..

[47] Mikaeloff Y., Jambaqué I., Hertz-Pannier L., Zamfirescu A., Adamsbaum C., Plouin P., Dulac O., Chiron C. (2006). Devastating epileptic encephalopathy in school-aged children (DESC): A pseudo encephalitis. Epilepsy Res..

[48] Naylor D.E., Liu H., Niquet J., Wasterlain C.G. (2013). Rapid surface accumulation of NMDA receptors increases glutamatergic excitation during status epilepticus. Neurobiol. Dis..

[49] Kapur J. (2018). Role of NMDA receptors in the pathophysiology and treatment of status epilepticus. Epilepsia Open.

[50] Garrido-Sanabria E.R., Otalora L.F., Arshadmansab M.F., Herrera B., Francisco S., Ermolinsky B.S. (2008). Impaired expression and function of group II metabotropic glutamate receptors in pilocarpine-treated chronically epileptic rats. Brain Res..

[51] Watanabe Y., Kaida Y., Fukuhara S., Takechi K., Uehara T., Kamei C. (2011). Participation of metabotropic glutamate receptors in pentetrazol-induced kindled seizure. Epilepsia.

[52] Bocchio M., Lukacs I.P., Stacey R., Plaha P., Apostolopoulos V., Livermore L., Sen A., Ansorge O., Gillies M.J., Somogyi P. (2018). Group II Metabotropic Glutamate Receptors Mediate Presynaptic Inhibition of Excitatory Transmission in Pyramidal Neurons of the Human Cerebral Cortex. Front. Cell. NeuroSci..

[53] Doyle S., Pyndiah S., De Gois S., Erickson J.D. (2010). Excitation-transcription coupling via calcium/calmodulin-dependent protein kinase/ERK1/2 signaling mediates the coordinate induction of VGLUT2 and Narp triggered by a prolonged increase in glutamatergic synaptic activity. J. Biol. Chem..

[54] Gangarossa G., Di Benedetto M., O’Sullivan G.J., Dunleavy M., Alcacer C., Bonito-Oliva A., Henshall D.C., Waddington J.L., Valjent E., Fisone G. (2011). Convulsant doses of a dopamine D1 receptor agonist result in Erk-dependent increases in Zif268 and Arc/Arg3.1 expression in mouse dentate gyrus. PLoS ONE.

[55] Gangarossa G., Castell L., Castro L., Tarot P., Veyrunes F., Vincent P., Bertaso F., Valjent E. (2019). Contrasting patterns of ERK activation in the tail of the striatum in response to aversive and rewarding signals. J. NeuroChem..

[56] Nateri A.S., Raivich G., Gebhardt C., Da Costa C., Naumann H., Vreugdenhil M., Makwana M., Brandner S., Adams R.H., Jefferys J.G. (2007). ERK activation causes epilepsy by stimulating NMDA receptor activity. EMBO J..

[57] Pernice H.F., Schieweck R., Kiebler M.A., Popper B. (2016). mTOR and MAPK: From localized translation control to epilepsy. BMC NeuroSci..

[58] Lin T.Y., Lu C.W., Wang C.C., Lu J.F., Wang S.J. (2012). Hispidulin inhibits the release of glutamate in rat cerebrocortical nerve terminals. Toxicol. Appl. Pharmacol..

[59] Glazova M.V., Nikitina L.S., Hudik K.A., Kirillova O.D., Dorofeeva N.A., Korotkov A.A., Chernigovskaya E.V. (2015). Inhibition of ERK1/2 signaling prevents epileptiform behavior in rats prone to audiogenic seizures. J. NeuroChem..

[60] Bozzi Y., Borrelli E. (2013). The role of dopamine signaling in epileptogenesis. Front. Cell NeuroSci..

[61] Beaulieu J.M., Gainetdinov R.R. (2011). The physiology, signaling, and pharmacology of dopamine receptors. Pharmacol. Rev..

[62] Yakushev I.Y., Dupont E., Buchholz H.G., Tillmanns J., Debus F., Cumming P., Heimann A., Fellgiebel A., Luhmann H.J., Landvogt C. (2010). In vivo imaging of dopamine receptors in a model of temporal lobe epilepsy. Epilepsia.

[63] Bernedo Paredes V.E., Buchholz H.G., Gartenschläger M., Breimhorst M., Schreckenberger M., Werhahn K.J. (2015). Reduced D2/D3 Receptor Binding of Extrastriatal and Striatal Regions in Temporal Lobe Epilepsy. PLoS ONE.

[64] Dorofeeva N.A., Grigorieva Y.S., Nikitina L.S., Lavrova E.A., Nasluzova E.V., Glazova M.V., Chernigovskaya E.V. (2017). Effects of ERK1/2 kinases inactivation on the nigrostriatal system of Krushinsky-Molodkina rats genetically prone to audiogenic seizures. Neurol. Res..

[65] Svob Strac D., Pivac N., Smolders I.J., Fogel W.A., De Deurwaerdere P., Di Giovanni G. (2016). Monoaminergic Mechanisms in Epilepsy May Offer Innovative Therapeutic Opportunity for Monoaminergic Multi-Target Drugs. Front. NeuroSci..

[66] Medvedev A.E., Rajgorodskaya D.I., Gorkin V.Z., Fedotova I.B., Semiokhina A.F. (1992). The role of lipid peroxidation in the possible involvement of membrane-bound monoamine oxidases in gamma-aminobutyric acid and glucosamine deamination in rat brain. Focus on chemical pathogenesis of experimental audiogenic epilepsy. Mol. Chem. Neuropathol..

[67] Dailey J.W., Yan Q.S., Adams-Curtis L.E., Ryu J.R., Ko K.H., Mishra P.K., Jobe P.C. (1996). Neurochemical correlates of antiepileptic drugs in the genetically epilepsy-prone rat (GEPR). Life Sci..

[68] Chauvel P., Trottier S. (1986). Role of noradrenergic ascending system in extinction of epileptic phenomena. Adv. Neurol..

[69] Chauvel P., Trottier S., Nassif S., Dedek J. (1982). Is an alteration of noradrenergic afferents involved in focal epilepsies?. Rev. Electroencephalogr. Neuro. Physiol. Clin..

[70] Sourbron J., Lagae L. (2022). Serotonin receptors in epilepsy: Novel treatment targets?. Epilepsia Open.

[71] Jerlicz M., Kostowski W., Bidziński A., Hauptman M., Dymecki J. (1978). Audiogenic seizures in rats: Relation to noradrenergic neurons of the locus coeruleus. Acta Physiol. Pol..

[72] Fritschy J.M. (2008). Epilepsy, E/I Balance and GABA (A) Receptor Plasticity. Front. Mol. NeuroSci..

[73] Morimoto K., Sato H., Yamamoto Y., Watanabe T., Suwaki H. (1997). Antiepileptic effects of tiagabine, a selective GABA uptake inhibitor, in the rat kindling model of temporal lobe epilepsy. Epilepsia.

[74] Fueta Y., Vasilets L.A., Takeda K., Kawamura M., Schwarz W. (2003). Down-regulation of GABA-transporter function by hippocampal translation products: Its possible role in epilepsy. NeuroScience.

[75] Cai K., Wang J., Eissman J., Nwosu G., Shen W., Liang H.C., Li X.J., Zhu H.X., Yi Y.H., Song J. (2019). A missense mutation in SLC6A1 associated with Lennox-Gastaut syndrome impairs GABA transporter 1 protein trafficking and function. Exp. Neurol..

[76] Shaye H., Stauch B., Gati C., Cherezov V. (2021). Molecular mechanisms of metabotropic GABA. Sci. Adv..

[77] Zafar S., Jabeen I. (2018). Structure, Function, and Modulation of γ-Aminobutyric Acid Transporter 1 (GAT1) in Neurological Disorders: A Pharmacoinformatic Prospective. Front. Chem..

[78] Quilichini P.P., Chiron C., Ben-Ari Y., Gozlan H. (2006). Stiripentol, a putative antiepileptic drug, enhances the duration of opening of GABA-A receptor channels. Epilepsia.

[79] Catterall W.A., Kalume F., Oakley J.C. (2010). NaV1.1 channels and epilepsy. J. Physiol..

[80] Kardos J., Szabó Z., Héja L. (2016). Framing Neuro-Glia Coupling in Antiepileptic Drug Design. J. Med. Chem..

[81] Héja L., Nyitrai G., Kékesi O., Dobolyi A., Szabó P., Fiáth R., Ulbert I., Pál-Szenthe B., Palkovits M., Kardos J. (2012). Astrocytes convert network excitation to tonic inhibition of neurons. BMC Biol..

[82] Löscher W., Gillard M., Sands Z.A., Kaminski R.M., Klitgaard H. (2016). Synaptic Vesicle Glycoprotein 2A Ligands in the Treatment of Epilepsy and Beyond. CNS Drugs.

[83] Seifert G., Carmignoto G., Steinhäuser C. (2010). Astrocyte dysfunction in epilepsy. Brain Res Rev..

[84] Patel D.C., Tewari B.P., Chaunsali L., Sontheimer H. (2019). Neuron-glia interactions in the pathophysiology of epilepsy. Nat. Rev. NeuroSci..

[85] Heuser K., Szokol K., Taubøll E. (2014). The role of glial cells in epilepsy. Tidsskr. Nor. Laegeforen..

[86] Kaila K., Ruusuvuori E., Seja P., Voipio J., Puskarjov M. (2014). GABA actions and ionic plasticity in epilepsy. Curr. Opin. Neurobiol..

[87] Rana A., Musto A.E. (2018). The role of inflammation in the development of epilepsy. J. Neuroinflamm..

[88] Vezzani A., French J., Bartfai T., Baram T.Z. (2011). The role of inflammation in epilepsy. Nat. Rev. Neurol..

[89] Avakyan G., Avakyan G. (2017). Transformation of the epileptic system. Current status of the problem and possible ways to solve it. Epilepsy Paroxysmal Cond..

[90] Verma I.C., Bhatia S., Arora V. (2021). Genetic Testing in Pediatric Epilepsy. Indian J. Pediatr..

[91] Steinlein O.K. (2004). Genes and mutations in human idiopathic epilepsy. Brain Dev..

[92] Kjeldsen M.J., Corey L.A., Christensen K., Friis M.L. (2003). Epileptic seizures and syndromes in twins: The importance of genetic factors. Epilepsy Res..

[93] Berkovic S.F., Howell R.A., Hay D.A., Hopper J.L. (1998). Epilepsies in twins: Genetics of the major epilepsy syndromes. Ann. Neurol..

[94] Kjeldsen M.J., Corey L.A., Solaas M.H., Friis M.L., Harris J.R., Kyvik K.O., Christensen K., Pellock J.M. (2005). Genetic factors in seizures: A population-based study of 47,626 US, Norwegian and Danish twin pairs. Twin Res. Hum. Genet..

[95] Sugawara T., Tsurubuchi Y., Agarwala K.L., Ito M., Fukuma G., Mazaki-Miyazaki E., Nagafuji H., Noda M., Imoto K., Wada K. (2001). A missense mutation of the Na+ channel alpha II subunit gene Na(v)1.2 in a patient with febrile and afebrile seizures causes channel dysfunction. Proc. Natl. Acad. Sci. USA.

[96] Falace A., Filipello F., La Padula V., Vanni N., Madia F., De Pietri Tonelli D., de Falco F.A., Striano P., Dagna Bricarelli F., Minetti C. (2010). TBC1D24, an ARF6-interacting protein, is mutated in familial infantile myoclonic epilepsy. Am. J. Hum. Genet..

[97] Fehr S., Wilson M., Downs J., Williams S., Murgia A., Sartori S., Vecchi M., Ho G., Polli R., Psoni S. (2013). The CDKL5 disorder is an independent clinical entity associated with early-onset encephalopathy. Eur. J. Hum. Genet..

[98] Zhou Q., Wang J., Xia L., Li R., Zhang Q., Pan S. (2021). SYN1 Mutation Causes X-Linked Toothbrushing Epilepsy in a Chinese Family. Front. Neurol..

[99] Garcia C.C., Blair H.J., Seager M., Coulthard A., Tennant S., Buddles M., Curtis A., Goodship J.A. (2004). Identification of a mutation in synapsin I, a synaptic vesicle protein, in a family with epilepsy. J. Med. Genet..

[100] Thakran S., Guin D., Singh P., Kukal S., Rawat C., Yadav S., Kushwaha S.S., Srivastava A.K., Hasija Y., Saso L. (2020). Genetic Landscape of Common Epilepsies: Advancing towards Precision in Treatment. Int. J. Mol. Sci..

[101] Ferraro T.N., Buono R.J. (2006). Polygenic epilepsy. Adv. Neurol..

[102] Koeleman B.P.C. (2018). What do genetic studies tell us about the heritable basis of common epilepsy? Polygenic or complex epilepsy?. NeuroSci. Lett.

[103] Leu C., Richardson T.G., Kaufmann T., van der Meer D., Andreassen O.A., Westlye L.T., Busch R.M., Davey Smith G., Lal D. (2020). Pleiotropy of polygenic factors associated with focal and generalized epilepsy in the general population. PLoS ONE.

[104] Farnaes L., Nahas S.A., Chowdhury S., Nelson J., Batalov S., Dimmock D.M., Kingsmore S.F., Investigators R. (2017). Rapid whole-genome sequencing identifies a novel. Mol. Case Stud..

[105] Kodera H., Ohba C., Kato M., Maeda T., Araki K., Tajima D., Matsuo M., Hino-Fukuyo N., Kohashi K., Ishiyama A. (2016). De novo GABRA1 mutations in Ohtahara and West syndromes. Epilepsia.

[106] Cossette P., Lortie A., Vanasse M., Saint-Hilaire J.M., Rouleau G.A. (2005). Autosomal dominant juvenile myoclonic epilepsy and GABRA1. Adv. Neurol..

[107] Belousova E.D., Zavadenko N.N., Kholin A.A., Sharkov A.A. (2017). New classifications of epilepsies and seizure types created by the International League against Epilepsy. Zhurnal Nevrol. Psikhiatrii Im. SS Korsakova.

[108] Nakayama T., Ishii A., Yoshida T., Nasu H., Shimojima K., Yamamoto T., Kure S., Hirose S. (2018). Somatic mosaic deletions involving SCN1A cause Dravet syndrome. Am. J. Med. Genet. A.

[109] Guerrini R., Marini C., Mantegazza M. (2014). Genetic epilepsy syndromes without structural brain abnormalities: Clinical features and experimental models. Neurotherapeutics.

[110] Liu X.-X., Yang L., Shao L.-X., He Y., Wu G., Bao Y.-H., Lu N.-N., Gong D.-M., Lu Y.-P., Cui T.-T. (2019). Endothelial Cdk5 deficit leads to the development of spontaneous epilepsy through CXCL1/CXCR2-mediated reactive astrogliosis. J. Exp. Med..

[111] Breuss M.W., Sultan T., James K.N., Rosti R.O., Scott E., Musaev D., Furia B., Reis A., Sticht H., Al-Owain M. (2016). Autosomal-Recessive Mutations in the tRNA Splicing Endonuclease Subunit TSEN15 Cause Pontocerebellar Hypoplasia and Progressive Microcephaly. Am. J. Hum. Genet..

[112] Poulton C., Oegema R., Heijsman D., Hoogeboom J., Schot R., Stroink H., Willemsen M.A., Verheijen F.W., Van De Spek P., Kremer A. (2012). Progressive cerebellar atrophy and polyneuropathy: Expanding the spectrum of PNKP mutations. Neurogenetics.

[113] Liu J.S. (2011). Molecular Genetics of Neuronal Migration Disorders. Curr. Neurol. Neurosci. Rep..

[114] Finsterer J. (2004). Mitochondriopathies. Eur. J. Neurol..

[115] Lim A., Thomas R.H. (2020). The mitochondrial epilepsies. Eur. J. Paediatr. Neurol..

[116] Musto E., Gardella E., Møller R.S. (2020). Recent advances in treatment of epilepsy-related sodium channelopathies. Eur. J. Paediatr. Neurol..

[117] Zhu X., Han X., Blendy J.A., Porter B.E. (2012). Decreased CREB levels suppress epilepsy. Neurobiol. Dis..

[118] Switon K., Kotulska K., Janusz-Kaminska A., Zmorzynska J., Jaworski J. (2017). Molecular neurobiology of mTOR. NeuroScience.

[119] Robison A.J. (2014). Emerging role of CaMKII in neuropsychiatric disease. Trends NeuroSci..

[120] Liu J., Reeves C., Michalak Z., Coppola A., Diehl B., Sisodiya S.M., Thom M. (2014). Evidence for mTOR pathway activation in a spectrum of epilepsy-associated pathologies. Acta Neuropathol. Commun..

[121] Chuvakova L.N., Funikov S.Y., Rezvykh A.P., Davletshin A.I., Evgen’ev M.B., Litvinova S.A., Fedotova I.B., Poletaeva I.I., Garbuz D.G. (2021). Transcriptome of the Krushinsky-Molodkina Audiogenic Rat Strain and Identification of Possible Audiogenic Epilepsy-Associated Genes. Front. Mol. NeuroSci..

[122] Venediktova N.I., Gorbacheva O.S., Belosludtseva N.V., Fedotova I.B., Surina N.M., Poletaeva I.I., Kolomytkin O.V., Mironova G.D. (2017). Energetic, oxidative and ionic exchange in rat brain and liver mitochondria at experimental audiogenic epilepsy (Krushinsky–Molodkina model). J. Bioenerg. Biomembr..

[123] Zavala-Tecuapetla C., Cuellar-Herrera M., Luna-Munguia H. (2020). Insights into Potential Targets for Therapeutic Intervention in Epilepsy. Int. J. Mol. Sci..

[124] Sanz P., Garcia-Gimeno M.A. (2020). Reactive Glia Inflammatory Signaling Pathways and Epilepsy. Int. J. Mol. Sci..

[125] Dixit A.B., Banerjee J., Tripathi M., Sarkar C., Chandra P.S. (2017). Synaptic roles of cyclin-dependent kinase 5 & its implications in epilepsy. Indian J. Med. Res..

[126] Crino P.B. (2015). mTOR Signaling in Epilepsy: Insights from Malformations of Cortical Development. Cold Spring Harb. Perspect. Med..

[127] Kandratavicius L., Balista P.A., Lopes-Aguiar C., Ruggiero R.N., Umeoka E.H., Garcia-Cairasco N., Bueno-Junior L.S., Leite J.P. (2014). Animal models of epilepsy: Use and limitations. Neuropsychiatr. Dis. Treat..

[128] McNamara J.O., Bonhaus D.W., Shin C., Crain B.J., Gellman R.L., Giacchino J.L. (1985). The kindling model of epilepsy: A critical review. CRC Crit. Rev. Clin. Neurobiol..

[129] Ferlazzo E., Gasparini S., Beghi E., Sueri C., Russo E., Leo A., Labate A., Gambardella A., Belcastro V., Striano P. (2016). Epilepsy in cerebrovascular diseases: Review of experimental and clinical data with meta-analysis of risk factors. Epilepsia.

[130] Bonvallet M., Dell P., Hugelin A. (1952). Olfactory, gustatory, visceral, vagal, visual and auditory projections in the grey formations of the forebrain of the cat. J. Physiol..

[131] Kopeloff L.M. (1960). Experimental epilepsy in the mouse. Proc. Soc. Exp. Biol. Med..

[132] Gorter J.A., van Vliet E.A., Lopes da Silva F.H. (2016). Which insights have we gained from the kindling and post-status epilepticus models?. J. NeuroSci. Methods.

[133] Castel-Branco M.M., Alves G.L., Figueiredo I.V., Falcão A.C., Caramona M.M. (2009). The maximal electroshock seizure (MES) model in the preclinical assessment of potential new antiepileptic drugs. Methods Find. Exp. Clin. Pharmacol..

[134] Yuen E.S., Trocóniz I.F. (2015). Can pentylenetetrazole and maximal electroshock rodent seizure models quantitatively predict antiepileptic efficacy in humans?. Seizure.

[135] De Sarro G., Russo E., Citraro R., Meldrum B.S. (2017). Genetically epilepsy-prone rats (GEPRs) and DBA/2 mice: Two animal models of audiogenic reflex epilepsy for the evaluation of new generation AEDs. Epilepsy Behav..

[136] Sarkisova K., van Luijtelaar G. (2010). The WAG/Rij strain: A genetic animal model of absence epilepsy with comorbidity of depression [corrected]. Prog. Neuro-Psychopharmacol. Biol. Psychiatry.

[137] Seyfried T.N. (1979). Audiogenic seizures in mice. Fed. Proc..

[138] Maxson S.C. (2017). A genetic context for the study of audiogenic seizures. Epilepsy Behav..

[139] Fedotova I.B., Surina N.M., Nikolaev G.M., Revishchin A.V., Poletaeva I.I. (2021). Rodent Brain Pathology, Audiogenic Epilepsy. Biomedicines.

[140] A Fless D., Salimov R.M. (1974). An analysis of the phase of prespasmodic motor excitation in rats with audiogenic seizures. Bull. Exp. Biol. Med..

[141] Salimov R.M., Fless D.A. (1977). Influence of convulsive and pre-convulsive components of an audiogenic seizure on the process of consolidation of temporary connections. Bull. Exp. Biol. Med..

[142] Poletaeva I.I., Surina N.M., Kostina Z.A., Perepelkina O.V., Fedotova I.B. (2017). The Krushinsky-Molodkina rat strain: The study of audiogenic epilepsy for 65years. Epilepsy Behav..

[143] Reigel C.E., Dailey J.W., Jobe P.C. (1986). The genetically epilepsy-prone rat: An overview of seizure-prone characteristics and responsiveness to anticonvulsant drugs. Life Sci..

[144] Zhao D.Y., Wu X.R., Pei Y.Q., Zuo Q.H. (1985). Kindling phenomenon of hyperthermic seizures in the epilepsy-prone versus the epilepsy-resistant rat. Brain Res..

[145] Bo X., Zhiguo W., Xiaosu Y., Guoliang L., Guangjie X. (2002). Analysis of gene expression in genetic epilepsy-prone rat using a cDNA expression array. Seizure.

[146] Muñoz L.J., Carballosa-Gautam M.M., Yanowsky K., García-Atarés N., López D.E. (2017). The genetic audiogenic seizure hamster from Salamanca: The GASH:Sal. Epilepsy Behav..

[147] Dolina S., Peeling J., Sutherland G., Pillay N., Greenberg A. (1993). Effect of sustained pyridoxine treatment on seizure susceptibility and regional brain amino acid levels in genetically epilepsy-prone BALB/c mice. Epilepsia.

[148] Martin B., Dieuset G., Pawluski J.L., Costet N., Biraben A. (2020). Audiogenic seizure as a model of sudden death in epilepsy: A comparative study between four inbred mouse strains from early life to adulthood. Epilepsia.

[149] Poletaeva I.I., Fedotova I.B., Sourina N.M., Kostina Z.A. (2011). Audiogenic seizures-biological phenomenon and experimental model of human epilepsies. Clin. Genet. Asp. Epilepsy.

[150] Laird H.E., Dailey J.W., Jobe P.C. (1984). Neurotransmitter abnormalities in genetically epileptic rodents. Fed. Proc..

[151] Mishra P.K., Kahle E.H., Bettendorf A.F., Dailey J.W., Jobe P.C. (1993). Anticonvulsant effects of intracerebroventricularly administered norepinephrine are potentiated in the presence of monoamine oxidase inhibition in severe seizure genetically epilepsy-prone rats (GEPR-9s). Life Sci..

[152] Kash S.F., Johnson R.S., Tecott L.H., Noebels J.L., Mayfield R.D., Hanahan D., Baekkeskov S. (1997). Epilepsy in mice deficient in the 65-kDa isoform of glutamic acid decarboxylase. Proc. Natl. Acad. Sci. USA.

[153] N’Gouemo P., Faingold C.L. (1998). Periaqueductal gray neurons exhibit increased responsiveness associated with audiogenic seizures in the genetically epilepsy-prone rat. NeuroScience.

[154] N’Gouemo P., Faingold C.L. (1999). The periaqueductal grey is a critical site in the neuronal network for audiogenic seizures: Modulation by GABA(A), NMDA and opioid receptors. Epilepsy Res..

[155] Willott J.F., Lu S.M. (1980). Midbrain pathways of audiogenic seizures in DBA/2 mice. Exp. Neurol..

[156] Leite J.P., Garcia-Cairasco N., Cavalheiro E.A. (2002). New insights from the use of pilocarpine and kainate models. Epilepsy Res..

[157] Garcia-Cairasco N., Sabbatini R.M. (1991). Possible interaction between the inferior colliculus and the substantia nigra in audiogenic seizures in Wistar rats. Physiol. Behav..

[158] Simler S., Hirsch E., Danober L., Motte J., Vergnes M., Marescaux C. (1994). C-fos expression after single and kindled audiogenic seizures in Wistar rats. NeuroSci. Lett..

[159] Browning R.A., Wang C., Nelson D.K., Jobe P.C. (1999). Effect of precollicular transection on audiogenic seizures in genetically epilepsy-prone rats. Exp. Neurol..

[160] Ribak C.E., Manio A.L., Navetta M.S., Gall C.M. (1997). In situ hybridization for c-fos mRNA reveals the involvement of the superior colliculus in the propagation of seizure activity in genetically epilepsy-prone rats. Epilepsy Res..

[161] Ribak C.E., Morin C.L. (1995). The role of the inferior colliculus in a genetic model of audiogenic seizures. Anat. Embryol..

[162] Sánchez-Benito D., Gómez-Nieto R., Hernández-Noriega S., Murashima A.A.B., de Oliveira J.A.C., Garcia-Cairasco N., López D.E., Hyppolito M.A. (2017). Morphofunctional alterations in the olivocochlear efferent system of the genetic audiogenic seizure-prone hamster GASH:Sal. Epilepsy Behav..

[163] Zivanovic D., Stanojlovic O., Mirkovic S., Susic V. (2005). Ontogenetic study of metaphit-induced audiogenic seizures in rats. Brain Res. Dev. Brain Res..

[164] Susic V., Reith M.E., Zlokovic B.V., Lajtha A., Jacobson A.E., Rice K.C., Lipovac M.N. (1991). Electroencephalographic characteristics of audiogenic seizures induced in metaphit-treated small rodents. Epilepsia.

[165] 165 Stanojlović O., Zivanović D., Mirković S., Vucević D. (2004). Behavioral and electroencephalographic effects of delta sleep inducing peptide and its analogue on metaphit-induced audiogenic seizures in rats. Srp. Arh. Celok. Lek..

[166] Kovalzon V.M., Strekalova T.V. (2006). Delta sleep-inducing peptide (DSIP): A still unresolved riddle. J. Neurochem..

[167] Tukhovskaya E.A., Ismailova A.M., Shaykhutdinova E.R., Slashcheva G.A., Prudchenko I.A., Mikhaleva I.I., Khokhlova O.N., Murashev A.N., Ivanov V.T. (2021). Delta Sleep-Inducing Peptide Recovers Motor Function in SD Rats after Focal Stroke. Molecules.

[168] Khvatova E.M., Samartzev V.N., Zagoskin P.P., Prudchenko I.A., Mikhaleva I.I. (2003). Delta sleep inducing peptide (DSIP): Effect on respiration activity in rat brain mitochondria and stress protective potency under experimental hypoxia. Peptides.

[169] Gupta V., Awasthi N., Wagner B.J. (2007). Specific Activation of the Glucocorticoid Receptor and Modulation of Signal Transduction Pathways in Human Lens Epithelial Cells. Investig. Opthalmol. Vis. Sci..

[170] Fedotova I.B., Semiokhina A.F., Arkhipova G.V., Nikashin A.V., Burlakova E.B. (1988). Differences in the lipid composition of the brain of rats of the Krushinskiĭ-Molodkina strain during an audiogenic seizure attack and in myoclonus. Zhurnal Vyss. Nervn. Deiatelnosti Im. IP Pavlov..

[171] Baulac S., Ishida S., Mashimo T., Boillot M., Fumoto N., Kuwamura M., Ohno Y., Takizawa A., Aoto T., Ueda M. (2012). A rat model for LGI1-related epilepsies. Hum. Mol. Genet..

[172] Bassell G.J., Warren S.T. (2008). Fragile X syndrome: Loss of local mRNA regulation alters synaptic development and function. Neuron.

[173] Berry-Kravis E., Filipink R.A., Frye R.E., Golla S., Morris S.M., Andrews H., Choo T.H., Kaufmann W.E., Consortium F. (2021). Seizures in Fragile X Syndrome: Associations and Longitudinal Analysis of a Large Clinic-Based Cohort. Front. Pediatr..

[174] Musumeci S.A., Calabrese G., Bonaccorso C.M., D’Antoni S., Brouwer J.R., Bakker C.E., Elia M., Ferri R., Nelson D.L., Oostra B.A. (2007). Audiogenic seizure susceptibility is reduced in fragile X knockout mice after introduction of FMR1 transgenes. Exp. Neurol..

[175] Musumeci S.A., Bosco P., Calabrese G., Bakker C., De Sarro G.B., Elia M., Ferri R., Oostra B.A. (2000). Audiogenic seizures susceptibility in transgenic mice with fragile X syndrome. Epilepsia.

[176] Chen L., Toth M. (2001). Fragile X mice develop sensory hyperreactivity to auditory stimuli. NeuroScience.

[177] Pacey L.K., Tharmalingam S., Hampson D.R. (2011). Subchronic administration and combination metabotropic glutamate and GABAB receptor drug therapy in fragile X syndrome. J. Pharmacol. Exp. Ther..

[178] McMillan D.R., Kayes-Wandover K.M., Richardson J.A., White P.C. (2002). Very large G protein-coupled receptor-1, the largest known cell surface protein, is highly expressed in the developing central nervous system. J. Biol. Chem..

[179] Shin D., Lin S.T., Fu Y.H., Ptácek L.J. (2013). Very large G protein-coupled receptor 1 regulates myelin-associated glycoprotein via Gαs/Gαq-mediated protein kinases A/C. Proc. Natl. Acad. Sci. USA.

[180] Yagi H., Noguchi Y., Kitamura K., Sato M. (2009). Deficiency of Vlgr1 resulted in deafness and susceptibility to audiogenic seizures while the degree of hearing impairment was not correlated with seizure severity in C57BL/6- and 129-backcrossed lines of Vlgr1 knockout mice. NeuroSci. Lett..

[181] Damasceno S., Fonseca P.A.S., Rosse I.C., Moraes M.F.D., de Oliveira J.A.C., Garcia-Cairasco N., Brunialti Godard A.L. (2021). Putative Causal Variant on. Front. Neurol..

[182] Bednarek A.K., Laflin K.J., Daniel R.L., Liao Q., Hawkins K.A., Aldaz C.M. (2000). WWOX, a novel WW domain-containing protein mapping to human chromosome 16q23.3-24.1, a region frequently affected in breast cancer. Cancer Res..

[183] Repudi S., Steinberg D.J., Elazar N., Breton V.L., Aquilino M.S., Saleem A., Abu-Swai S., Vainshtein A., Eshed-Eisenbach Y., Vijayaragavan B. (2021). Neuronal deletion of Wwox, associated with WOREE syndrome, causes epilepsy and myelin defects. Brain.

[184] He J., Zhou W., Shi J., Zhang B., Wang H. (2020). A Chinese patient with epilepsy and WWOX compound heterozygous mutations. Epileptic Disord..

[185] Suzuki H., Katayama K., Takenaka M., Amakasu K., Saito K., Suzuki K. (2009). A spontaneous mutation of the Wwox gene and audiogenic seizures in rats with lethal dwarfism and epilepsy. Genes Brain Behav..

[186] Knowles J.K., Xu H., Soane C., Batra A., Saucedo T., Frost E., Tam L.T., Fraga D., Ni L., Villar K. (2022). Maladaptive myelination promotes generalized epilepsy progression. Nat. NeuroSci..

[187] Repudi S., Kustanovich I., Abu-Swai S., Stern S., Aqeilan R.I. (2021). Neonatal neuronal WWOX gene therapy rescues Wwox null phenotypes. EMBO Mol. Med..

[188] Brennan T.J., Seeley W.W., Kilgard M., Schreiner C.E., Tecott L.H. (1997). Sound-induced seizures in serotonin 5-HT2c receptor mutant mice. Nat. Genet..

[189] Celada P., Puig M.V., Artigas F. (2013). Serotonin modulation of cortical neurons and networks. Front. Integr. NeuroSci..

[190] Jiang X., Xing G., Yang C., Verma A., Zhang L., Li H. (2009). Stress impairs 5-HT2A receptor-mediated serotonergic facilitation of GABA release in juvenile rat basolateral amygdala. Neuropsychopharmacology.

[191] Crunelli V., Di Giovanni G. (2015). Differential Control by 5-HT and 5-HT1A, 2A, 2C Receptors of Phasic and Tonic GABAA Inhibition in the Visual Thalamus. CNS NeuroSci. Ther..

[192] Bagdy G., Kecskemeti V., Riba P., Jakus R. (2007). Serotonin and epilepsy. J. NeuroChem..

[193] Jakus R., Graf M., Juhasz G., Gerber K., Levay G., Halasz P., Bagdy G. (2003). 5-HT2C receptors inhibit and 5-HT1A receptors activate the generation of spike-wave discharges in a genetic rat model of absence epilepsy. Exp. Neurol..

[194] Srivastava S., Cohen J., Pevsner J., Aradhya S., McKnight D., Butler E., Johnston M., Fatemi A. (2014). A novel variant in GABRB2 associated with intellectual disability and epilepsy. Am. J. Med. Genet. A.

[195] Yang Y., Xiangwei W., Zhang X., Xiao J., Chen J., Yang X., Jia T., Yang Z., Jiang Y., Zhang Y. (2020). Phenotypic spectrum of patients with GABRB2 variants: From mild febrile seizures to severe epileptic encephalopathy. Dev. Med. Child Neurol..

[196] El Achkar C.M., Harrer M., Smith L., Kelly M., Iqbal S., Maljevic S., Niturad C.E., Vissers L.E.L.M., Poduri A., Yang E. (2021). Characterization of the GABRB2-Associated Neurodevelopmental Disorders. Ann. Neurol..

[197] Yeung R.K., Xiang Z.H., Tsang S.Y., Li R., Ho T.Y.C., Li Q., Hui C.K., Sham P.C., Qiao M.Q., Xue H. (2018). Gabrb2-knockout mice displayed schizophrenia-like and comorbid phenotypes with interneuron-astrocyte-microglia dysregulation. Transl. Psychiatry.

[198] Zhu Y.H., Liu H., Zhang L.Y., Zeng T., Song Y., Qin Y.R., Li L., Liu L., Li J., Zhang B. (2014). Downregulation of LGI1 promotes tumor metastasis in esophageal squamous cell carcinoma. Carcinogenesis.

[199] Cowell J.K. (2014). LGI1: From zebrafish to human epilepsy. Prog. Brain Res..

[200] Kinboshi M., Shimizu S., Mashimo T., Serikawa T., Ito H., Ikeda A., Takahashi R., Ohno Y. (2019). Down-Regulation of Astrocytic Kir4.1 Channels during the Audiogenic Epileptogenesis in. Int. J. Mol. Sci..

[201] Kunapuli P., Kasyapa C.S., Hawthorn L., Cowell J.K. (2004). LGI1, a putative tumor metastasis suppressor gene, controls in vitro invasiveness and expression of matrix metalloproteinases in glioma cells through the ERK1/2 pathway. J. Biol. Chem..

[202] Yamagata A., Fukai S. (2020). Insights into the mechanisms of epilepsy from structural biology of LGI1-ADAM22. Cell Mol. Life Sci..

[203] Senechal K.R., Thaller C., Noebels J.L. (2005). ADPEAF mutations reduce levels of secreted LGI1, a putative tumor suppressor protein linked to epilepsy. Hum. Mol. Genet..

[204] Chabrol E., Navarro V., Provenzano G., Cohen I., Dinocourt C., Rivaud-Péchoux S., Fricker D., Baulac M., Miles R., Leguern E. (2010). Electroclinical characterization of epileptic seizures in leucine-rich, glioma-inactivated 1-deficient mice. Brain.

[205] Manis A.D., Palygin O., Isaeva E., Levchenko V., LaViolette P.S., Pavlov T.S., Hodges M.R., Staruschenko A. (2021). Kcnj16 knockout produces audiogenic seizures in the Dahl salt-sensitive rat. JCI Insight.

[206] Staruschenko A., Hodges M.R., Palygin O. (2022). Kir5.1 channels: Potential role in epilepsy and seizure disorders. Am. J. Physiol. Cell Physiol..

[207] Misawa H., Sherr E.H., Lee D.J., Chetkovich D.M., Tan A., Schreiner C.E., Bredt D.S. (2002). Identification of a monogenic locus (jams1) causing juvenile audiogenic seizures in mice. J. NeuroSci..

[208] Drayton M., Noben-Trauth K. (2006). Mapping quantitative trait loci for hearing loss in Black Swiss mice. Hear. Res..

[209] Charizopoulou N., Lelli A., Schraders M., Ray K., Hildebrand M.S., Ramesh A., Srisailapathy C.R., Oostrik J., Admiraal R.J., Neely H.R. (2011). Gipc3 mutations associated with audiogenic seizures and sensorineural hearing loss in mouse and human. Nat. Commun..

[210] Garcia-Gomes M.S.A., Zanatto D.A., Galvis-Alonso O.Y., Mejia J., Antiorio A.T.F.B., Yamamoto P.K., Olivato M.C.M., Sandini T.M., Flório J.C., Lebrun I. (2020). Behavioral and neurochemical characterization of the spontaneous mutation tremor, a new mouse model of audiogenic seizures. Epilepsy Behav..

[211] Kim J.H., Roberts D.S., Hu Y., Lau G.C., Brooks-Kayal A.R., Farb D.H., Russek S.J. (2012). Brain-derived neurotrophic factor uses CREB and Egr3 to regulate NMDA receptor levels in cortical neurons. J. NeuroChem..

[212] Kaitsuka T., Kiyonari H., Shiraishi A., Tomizawa K., Matsushita M. (2018). Deletion of Long Isoform of Eukaryotic Elongation Factor 1Bδ Leads to Audiogenic Seizures and Aversive Stimulus-Induced Long-Lasting Activity Suppression in Mice. Front. Mol. NeuroSci..

[213] Kaitsuka T., Tomizawa K., Matsushita M. (2011). Transformation of eEF1Bδ into heat-shock response transcription factor by alternative splicing. EMBO Rep..

[214] Reuter M.S., Tawamie H., Buchert R., Hosny Gebril O., Froukh T., Thiel C., Uebe S., Ekici A.B., Krumbiegel M., Zweier C. (2017). Diagnostic Yield and Novel Candidate Genes by Exome Sequencing in 152 Consanguineous Families With Neurodevelopmental Disorders. JAMA Psychiatry.

[215] Gachon F., Fonjallaz P., Damiola F., Gos P., Kodama T., Zakany J., Duboule D., Petit B., Tafti M., Schibler U. (2004). The loss of circadian PAR bZip transcription factors results in epilepsy. Genes Dev..

[216] Falsaperla R., Corsello G. (2017). Pyridoxine dependent epilepsies: New therapeutical poInt of view. Ital. J. Pediatr..

[217] Rotaru D.C., Mientjes E.J., Elgersma Y. (2020). Angelman Syndrome: From Mouse Models to Therapy. NeuroScience.

[218] Gentile J.K., Tan W.H., Horowitz L.T., Bacino C.A., Skinner S.A., Barbieri-Welge R., Bauer-Carlin A., Beaudet A.L., Bichell T.J., Lee H.S. (2010). A neurodevelopmental survey of Angelman syndrome with genotype-phenotype correlations. J. Dev. Behav. Pediatr..

[219] Buiting K., Williams C., Horsthemke B. (2016). Angelman syndrome—Insights into a rare neurogenetic disorder. Nat. Rev. Neurol..

[220] Jiang Y.H., Armstrong D., Albrecht U., Atkins C.M., Noebels J.L., Eichele G., Sweatt J.D., Beaudet A.L. (1998). Mutation of the Angelman ubiquitin ligase in mice causes increased cytoplasmic p53 and deficits of contextual learning and long-term potentiation. Neuron.

[221] Sonzogni M., Wallaard I., Santos S.S., Kingma J., du Mee D., van Woerden G.M., Elgersma Y. (2018). A behavioral test battery for mouse models of Angelman syndrome: A powerful tool for testing drugs and novel. Mol. Autism.

[222] Neumann P.E., Collins R.L. (1992). Confirmation of the influence of a chromosome 7 locus on susceptibility to audiogenic seizures. Mamm. Genome.

[223] Ferraro T.N., Golden G.T., Smith G.G., Martin J.F., Lohoff F.W., Gieringer T.A., Zamboni D., Schwebel C.L., Press D.M., Kratzer S.O. (2004). Fine mapping of a seizure susceptibility locus on mouse Chromosome 1: Nomination of Kcnj10 as a causative gene. Mamm. Genome.

[224] Jensen T.P., Filoteo A.G., Knopfel T., Empson R.M. (2007). Presynaptic plasma membrane Ca2+ ATPase isoform 2a regulates excitatory synaptic transmission in rat hippocampal CA3. J. Physiol..

[225] Inyushin M., Kucheryavykh L.Y., Kucheryavykh Y.V., Nichols C.G., Buono R.J., Ferraro T.N., Skatchkov S.N., Eaton M.J. (2010). Potassium channel activity and glutamate uptake are impaired in astrocytes of seizure-susceptible DBA/2 mice. Epilepsia.

[226] Boiarshinova O.S., Malashenko A.M., Bizikoeva F.Z., Revishchin A.V., Lil’p I.G., Poletaeva I.I. (2009). The 1xC3 panel of recombinant inbred mouse strains. Presence or absence of pathologic neurologic traits of one of parental strains. Genetika.

[227] Thompson J.L., Carl F.G., Holmes G.L. (1991). Effects of age on seizure susceptibility in genetically epilepsy-prone rats (GEPR-9s). Epilepsia.

[228] Ribak C.E., Roberts R.C., Byun M.Y., Kim H.L. (1988). Anatomical and behavioral analyses of the inheritance of audiogenic seizures in the progeny of genetically epilepsy-prone and Sprague-Dawley rats. Epilepsy Res..

[229] Ribak C.E. (2017). An abnormal GABAergic system in the inferior colliculus provides a basis for audiogenic seizures in genetically epilepsy-prone rats. Epilepsy Behav..

[230] Damasceno S., Gómez-Nieto R., Garcia-Cairasco N., Herrero-Turrión M.J., Marín F., Lopéz D.E. (2020). Top Common Differentially Expressed Genes in the Epileptogenic Nucleus of Two Strains of Rodents Susceptible to Audiogenic Seizures: WAR and GASH/Sal. Front. Neurol..

[231] Damasceno S., Menezes N.B., Rocha C.S., Matos A.H.B., Vieira A.S., Moraes M.F.D., Martins A.S., Lopes-Cendes I., Godard A.L.B. (2018). Transcriptome of the Wistar audiogenic rat (WAR) strain following audiogenic seizures. Epilepsy Res..

[232] Díaz-Casado E., Gómez-Nieto R., de Pereda J.M., Muñoz L.J., Jara-Acevedo M., López D.E. (2020). Analysis of gene variants in the GASH/Sal model of epilepsy. PLoS ONE.

[233] Díaz-Rodríguez S.M., López-López D., Herrero-Turrión M.J., Gómez-Nieto R., Canal-Alonso A., Lopéz D.E. (2020). Inferior Colliculus Transcriptome After Status Epilepticus in the Genetically Audiogenic Seizure-Prone Hamster GASH/Sal. Front. NeuroSci..

[234] López-López D., Gómez-Nieto R., Herrero-Turrión M.J., García-Cairasco N., Sánchez-Benito D., Ludeña M.D., López D.E. (2017). Overexpression of the immediate-early genes Egr1, Egr2, and Egr3 in two strains of rodents susceptible to audiogenic seizures. Epilepsy Behav..

[235] Roberts D.S., Raol Y.H., Bandyopadhyay S., Lund I.V., Budreck E.C., Passini M.A., Passini M.J., Wolfe J.H., Brooks-Kayal A.R., Russek S.J. (2005). Egr3 stimulation of GABRA4 promoter activity as a mechanism for seizure-induced up-regulation of GABA(A) receptor alpha4 subunit expression. Proc. Natl. Acad. Sci. USA.

[236] Brooks-Kayal A.R., Shumate M.D., Jin H., Rikhter T.Y., Coulter D.A. (1998). Selective changes in single cell GABA(A) receptor subunit expression and function in temporal lobe epilepsy. Nat. Med..

[237] Ando Y., Coelho T., Berk J.L., Cruz M.W., Ericzon B.G., Ikeda S., Lewis W.D., Obici L., Planté-Bordeneuve V., Rapezzi C. (2013). Guideline of transthyretin-related hereditary amyloidosis for clinicians. Orphanet J. Rare Dis..

[238] Franco A., Bentes C., de Carvalho M., Pereira P., Pimentel J., Conceição I. (2016). Epileptic seizures as a presentation of central nervous system involvement in TTR Val30Met-FAP. J. Neurol..

[239] Zhou L., Tang X., Li X., Bai Y., Buxbaum J.N., Chen G. (2019). Identification of transthyretin as a novel interacting partner for the δ subunit of GABAA receptors. PLoS ONE.

[240] Stein T.D., Anders N.J., DeCarli C., Chan S.L., Mattson M.P., Johnson J.A. (2004). Neutralization of transthyretin reverses the neuroprotective effects of secreted amyloid precursor protein (APP) in APPSW mice resulting in tau phosphorylation and loss of hippocampal neurons: Support for the amyloid hypothesis. J. NeuroSci..

[241] Kajiwara K., Sunaga K., Tsuda T., Sugaya A., Sugaya E., Kimura M. (2008). Peony root extract upregulates transthyretin and phosphoglycerate mutase in mouse cobalt focus seizure. BioChem. Biophys. Res. Commun..

[242] Li X., Buxbaum J.N. (2011). Transthyretin and the brain re-visited: Is neuronal synthesis of transthyretin protective in Alzheimer’s disease?. Mol. Neurodegener..

[243] Siderovski D.P., Heximer S.P., Forsdyke D.R. (1994). A human gene encoding a putative basic helix-loop-helix phosphoprotein whose mRNA increases rapidly in cycloheximide-treated blood mononuclear cells. DNA Cell Biol..

[244] Gold S.J., Heifets B.D., Pudiak C.M., Potts B.W., Nestler E.J. (2002). Regulation of regulators of G protein signaling mRNA expression in rat brain by acute and chronic electroconvulsive seizures. J. NeuroChem..

[245] Milanesi E., Cucos C.A., Matias-Guiu J.A., Piñol-Ripoll G., Manda G., Dobre M., Cuadrado A. (2021). Reduced Blood. Front. Aging NeuroSci..

[246] Noe’ F., Nissinen J., Pitkänen A., Gobbi M., Sperk G., During M., Vezzani A. (2007). Gene therapy in epilepsy: The focus on NPY. Peptides.

[247] Ercegovac M., Jovic N., Sokic D., Savic-Radojevic A., Coric V., Radic T., Nikolic D., Kecmanovic M., Matic M., Simic T. (2015). GSTA1, GSTM1, GSTP1 and GSTT1 polymorphisms in progressive myoclonus epilepsy: A Serbian case-control study. Seizure.

[248] Prabha T.S., Kumaraswami K., Kutala V.K. (2016). Association of GSTT1 and GSTM1 polymorphisms in South Indian Epilepsy Patients. Indian J. Exp. Biol..

[249] Jiang X., Raju P.K., D’Avanzo N., Lachance M., Pepin J., Dubeau F., Mitchell W.G., Bello-Espinosa L.E., Pierson T.M., Minassian B.A. (2019). Both gain-of-function and loss-of-function de novo CACNA1A mutations cause severe developmental epileptic encephalopathies in the spectrum of Lennox-Gastaut syndrome. Epilepsia.

[250] Sander T., Hildmann T., Kretz R., Fürst R., Sailer U., Bauer G., Schmitz B., Beck-Mannagetta G., Wienker T.F., Janz D. (1997). Allelic association of juvenile absence epilepsy with a GluR5 kainate receptor gene (GRIK1) polymorphism. Am. J. Med. Genet..

[251] Wu M., Katti P., Zhao Y., Peoples R.W. (2019). Positions in the N-methyl-D-aspartate Receptor GluN2C Subunit M3 and M4 Domains Regulate Alcohol Sensitivity and Receptor Kinetics. Alcohol. Clin. Exp. Res..

[252] Venkateswaran S., Myers K.A., Smith A.C., Beaulieu C.L., Schwartzentruber J.A., Majewski J., Bulman D., Boycott K.M., Dyment D.A., Consortium F.C. (2014). Whole-exome sequencing in an individual with severe global developmental delay and intractable epilepsy identifies a novel, de novo GRIN2A mutation. Epilepsia.

[253] Gun-Bilgic D., Polat M. (2022). Analysis of the Pathogenic Variants of Genes Using a Gene Panel in Turkish Epilepsy Patients. Clin. Lab..

[254] Moore S.W., Fieggen K., Honey E., Zaahl M. (2016). Novel Zeb2 gene variation in the Mowat Wilson syndrome (MWS). J. Pediatr. Surg..

[255] Yamada Y., Nomura N., Yamada K., Matsuo M., Suzuki Y., Sameshima K., Kimura R., Yamamoto Y., Fukushi D., Fukuhara Y. (2014). The spectrum of ZEB2 mutations causing the Mowat-Wilson syndrome in Japanese populations. Am. J. Med. Genet. A.

[256] Singh N.A., Westenskow P., Charlier C., Pappas C., Leslie J., Dillon J., Anderson V.E., Sanguinetti M.C., Leppert M.F., Consortium B.P. (2003). KCNQ2 and KCNQ3 potassium channel genes in benign familial neonatal convulsions: Expansion of the functional and mutation spectrum. Brain.

[257] Gupta S., Gellert M., Yang W. (2011). Mechanism of mismatch recognition revealed by human MutSβ bound to unpaired DNA loops. Nat. Struct. Mol. Biol..

[258] Francisconi S., Codenotti M., Ferrari Toninelli G., Uberti D., Memo M. (2006). Mitochondrial dysfunction and increased sensitivity to excitotoxicity in mice deficient in DNA mismatch repair. J. NeuroChem..

[259] Rahman S. (2015). Pathophysiology of mitochondrial disease causing epilepsy and status epilepticus. Epilepsy Behav..

[260] Chernigovskaya E.V., Dorofeeva N.A., Nasluzova E.V., Kulikov A.A., Ovsyannikova V.V., Glazova M.V. (2018). Apoptosis and proliferation in the inferior colliculus during postnatal development and epileptogenesis in audiogenic Krushinsky-Molodkina rats. Epilepsy Behav..

[261] Chernigovskaya E.V., Korotkov A.A., Dorofeeva N.A., Gorbacheva E.L., Kulikov A.A., Glazova M.V. (2019). Delayed audiogenic seizure development in a genetic rat model is associated with overactivation of ERK1/2 and disturbances in glutamatergic signaling. Epilepsy Behav..

[262] Fedotova I.B., Kostyna Z.A., Surina N.M., Poletaeva I.I. (2012). Laboratory rat selection for the trait “The absence of audiogenic seizure proneness”. Genetika.

[263] Poletaeva I.I., Surina N.M., Ashapkin V.V., Fedotova I.B., Merzalov I.B., Perepelkina O.V., Pavlova G.V. (2014). Maternal methyl-enriched diet in rat reduced the audiogenic seizure proneness in progeny. Pharmacol. BioChem. Behav..

[264] Parker M.H., Smith-Swintosky V.L., McComsey D.F., Huang Y., Brenneman D., Klein B., Malatynska E., White H.S., Milewski M.E., Herb M. (2009). Novel, broad-spectrum anticonvulsants containing a sulfamide group: Advancement of N-((benzo[b]thien-3-yl)methyl)sulfamide (JNJ-26990990) into human clinical studies. J Med. Chem..

[265] Nishi T., Metcalf C.S., Fujimoto S., Hasegawa S., Miyamoto M., Sunahara E., Watanabe S., Kondo S., White H.S. (2022). Anticonvulsive properties of soticlestat, a novel cholesterol 24-hydroxylase inhibitor. Epilepsia.

[266] Alachkar A., Ojha S.K., Sadeq A., Adem A., Frank A., Stark H., Sadek B. (2020). Experimental Models for the Discovery of Novel Anticonvulsant Drugs: Focus on Pentylenetetrazole-Ind.duced Seizures and Associated Memory Deficits. Curr. Pharm. Des..

[267] Barker-Haliski M., Steve White H. (2020). Validated animal models for antiseizure drug (ASD) discovery: Advantages and potential pitfalls in ASD screening. Neuropharmacology.

[268] Singh T., Mishra A., Goel R.K. (2021). PTZ kindling model for epileptogenesis, refractory epilepsy, and associated comorbidities: Relevance and reliability. Metab. Brain Dis..

[269] Ohno Y., Ishihara S., Terada R., Serikawa T., Sasa M. (2010). Antiepileptogenic and anticonvulsive actions of levetiracetam in a pentylenetetrazole kindling model. Epilepsy Res..

[270] Vinogradova L.V., van Rijn C.M. (2008). Anticonvulsive and antiepileptogenic effects of levetiracetam in the audiogenic kindling model. Epilepsia.

[271] Kasteleijn-Nolst Trenité D.G., Hirsch E. (2003). Levetiracetam: Preliminary efficacy in generalized seizures. Epileptic Disord..

[272] Löscher W. (1984). Genetic animal models of epilepsy as a unique resource for the evaluation of anticonvulsant drugs. A review. Methods Find. Exp. Clin. Pharmacol..

[273] Klitgaard H., Matagne A., Lamberty Y. (2002). Use of epileptic animals for adverse effect testing. Epilepsy Res..

[274] Chung L., Bey A.L., Towers A.J., Cao X., Kim I.H., Jiang Y.H. (2018). Lovastatin suppresses hyperexcitability and seizure in Angelman syndrome model. Neurobiol. Dis..

[275] Heulens I., D’Hulst C., Van Dam D., De Deyn P.P., Kooy R.F. (2012). Pharmacological treatment of fragile X syndrome with GABAergic drugs in a knockout mouse model. Behav. Brain Res..

[276] Wildin J.D., Pleuvry B.J. (1992). Tolerance to the anticonvulsant effects of clobazam in mice. Neuropharmacology.

[277] Gatta E., Cupello A., Di Braccio M., Grossi G., Robello M., Scicchitano F., Russo E., De Sarro G. (2016). Anticonvulsive Activity in Audiogenic DBA/2 Mice of 1,4-Benzodiazepines and 1,5-Benzodiazepines with Different Activities at Cerebellar Granule Cell GABA. J. Mol. NeuroSci..

[278] Muzzi M., Coppi E., Pugliese A.M., Chiarugi A. (2013). Anticonvulsant effect of AMP by direct activation of adenosine A1 receptor. Exp. Neurol..

[279] De Sarro G., De Sarro A., Di Paola E.D., Bertorelli R. (1999). Effects of adenosine receptor agonists and antagonists on audiogenic seizure-sensible DBA/2 mice. Eur. J. Pharmacol..

[280] Sparks D.L., Buckholtz N.S. (1980). Effects of 6-methoxy-1,2,3,4-tetrahydro-beta-carboline (6-MeO-THbetaC) on audiogenic seizures in DBA/2J mice. Pharmacol. BioChem. Behav..

[281] Brown J.W., Moeller A., Schmidt M., Turner S.C., Nimmrich V., Ma J., Rueter L.E., van der Kam E., Zhang M. (2016). Anticonvulsant effects of structurally diverse GABA(B) positive allosteric modulators in the DBA/2J audiogenic seizure test: Comparison to baclofen and utility as a pharmacodynamic screening model. Neuropharmacology.

[282] Gareri P., Condorelli D., Belluardo N., Gratteri S., Ferreri G., Donato Di Paola E., De Sarro A., De Sarro G. (2004). Influence of carbenoxolone on the anticonvulsant efficacy of conventional antiepileptic drugs against audiogenic seizures in DBA/2 mice. Eur. J. Pharmacol..

[283] Russo E., Donato di Paola E., Gareri P., Siniscalchi A., Labate A., Gallelli L., Citraro R., De Sarro G. (2013). Pharmacodynamic potentiation of antiepileptic drugs’ effects by some HMG-CoA reductase inhibitors against audiogenic seizures in DBA/2 mice. Pharmacol. Res..

[284] Madsen K.K., Clausen R.P., Larsson O.M., Krogsgaard-Larsen P., Schousboe A., White H.S. (2009). Synaptic and extrasynaptic GABA transporters as targets for anti-epileptic drugs. J. NeuroChem..

[285] Quansah H., N’Gouemo P. (2014). Amiloride and SN-6 suppress audiogenic seizure susceptibility in genetically epilepsy-prone rats. CNS NeuroSci. Ther..

[286] De Sarro C., Tallarico M., Pisano M., Gallelli L., Citraro R., De Sarro G., Leo A. (2022). Liraglutide chronic treatment prevents development of tolerance to antiseizure effects of diazepam in genetically epilepsy prone rats. Eur. J. Pharmacol..

[287] Vinogradova L.V., Kuznetsova G.D., Shatskova A.B., van Rijn C.M. (2005). Vigabatrin in low doses selectively suppresses the clonic component of audiogenically kindled seizures in rats. Epilepsia.

[288] François J., Boehrer A., Nehlig A. (2008). Effects of carisbamate (RWJ-333369) in two models of genetically determined generalized epilepsy, the GAERS and the audiogenic Wistar AS. Epilepsia.

[289] Damasceno D.D., Ferreira A.J., Doretto M.C., Almeida A.P. (2012). Anticonvulsant and antiarrhythmic effects of nifedipine in rats prone to audiogenic seizures. Braz J. Med. Biol. Res..

[290] Lazarini-Lopes W., Do Val-da Silva R.A., da Silva-Júnior R.M.P., Silva-Cardoso G.K., Leite-Panissi C.R.A., Leite J.P., Garcia-Cairasco N. (2021). Chronic cannabidiol (CBD) administration induces anticonvulsant and antiepileptogenic effects in a genetic model of epilepsy. Epilepsy Behav..

[291] Barrera-Bailón B., Oliveira J.A.C., López D.E., Muñoz L.J., Garcia-Cairasco N., Sancho C. (2017). Pharmacological and neuroethological study of the acute and chronic effects of lamotrigine in the genetic audiogenic seizure hamster (GASH:Sal). Epilepsy Behav..

[292] Barrera-Bailón B., Oliveira J.A., López D.E., Muñoz L.J., Garcia-Cairasco N., Sancho C. (2013). Pharmacological and neuroethological studies of three antiepileptic drugs in the Genetic Audiogenic Seizure Hamster (GASH:Sal). Epilepsy Behav..

[293] Turner T.J., Zourray C., Schorge S., Lignani G. (2021). Recent advances in gene therapy for neurodevelopmental disorders with epilepsy. J. NeuroChem..

[294] Nadadhur A.G., Alsaqati M., Gasparotto L., Cornelissen-Steijger P., van Hugte E., Dooves S., Harwood A.J., Heine V.M. (2019). Neuron-Glia Interactions Increase Neuronal Phenotypes in Tuberous Sclerosis Complex Patient iPSC-Derived Models. Stem Cell Rep..

[295] Schauwecker P.E. (2011). The relevance of individual genetic background and its role in animal models of epilepsy. Epilepsy Res..

[296] Schauwecker P.E. (2002). Complications associated with genetic background effects in models of experimental epilepsy. Prog Brain Res..

[297] Combi R., Dalprà L., Ferini-Strambi L., Tenchini M.L. (2005). Frontal lobe epilepsy and mutations of the corticotropin-releasing hormone gene. Ann. Neurol..

[298] Alakurtti K., Virtaneva K., Joensuu T., Palvimo J.J., Lehesjoki A.E. (2000). Characterization of the cystatin B gene promoter harboring the dodecamer repeat expanded in progressive myoclonus epilepsy, EPM1. Gene.

[299] Pathak S., Miller J., Morris E.C., Stewart W.C.L., Greenberg D.A. (2018). DNA methylation of the BRD2 promoter is associated with juvenile myoclonic epilepsy in Caucasians. Epilepsia.

[300] Bartel D.P. (2004). MicroRNAs: Genomics, biogenesis, mechanism, and function. Cell.

[301] Pitkänen A., Löscher W., Vezzani A., Becker A.J., Simonato M., Lukasiuk K., Gröhn O., Bankstahl J.P., Friedman A., Aronica E. (2016). Advances in the development of biomarkers for epilepsy. Lancet Neurol..

[302] Li M.M., Li X.M., Zheng X.P., Yu J.T., Tan L. (2014). MicroRNAs dysregulation in epilepsy. Brain Res..

[303] Ma Y. (2018). The Challenge of microRNA as a Biomarker of Epilepsy. Curr. NeuroPharmacol..

[304] Henshall D.C., Hamer H.M., Pasterkamp R.J., Goldstein D.B., Kjems J., Prehn J.H.M., Schorge S., Lamottke K., Rosenow F. (2016). MicroRNAs in epilepsy: Pathophysiology and clinical utility. Lancet Neurol..

[305] Karnati H.K., Panigrahi M.K., Gutti R.K., Greig N.H., Tamargo I.A. (2015). miRNAs: Key Players in Neurodegenerative Disorders and Epilepsy. J. Alzheimers Dis..

[306] Liu S., Fan M., Ma M., Ge J., Chen F. (2022). Long noncoding RNAs: Potential therapeutic targets for epilepsy. Front. NeuroSci..

